# Voting contagion: Modeling and analysis of a century of U.S. presidential elections

**DOI:** 10.1371/journal.pone.0177970

**Published:** 2017-05-18

**Authors:** Dan Braha, Marcus A. M. de Aguiar

**Affiliations:** 1 New England Complex Systems Institute, Cambridge, Massachusetts, United States of America; 2 University of Massachusetts, Dartmouth, Massachusetts, United States of America; 3 Universidade Estadual de Campinas, Campinas, São Paulo, Brazil; Universidad Nacional de Mar del Plata, ARGENTINA

## Abstract

Social influence plays an important role in human behavior and decisions. Sources of influence can be divided as external, which are independent of social context, or as originating from peers, such as family and friends. An important question is how to disentangle the social contagion by peers from external influences. While a variety of experimental and observational studies provided insight into this problem, identifying the extent of contagion based on large-scale observational data with an unknown network structure remains largely unexplored. By bridging the gap between the large-scale complex systems perspective of collective human dynamics and the detailed approach of social sciences, we present a parsimonious model of social influence, and apply it to a central topic in political science—elections and voting behavior. We provide an analytical expression of the county vote-share distribution, which is in excellent agreement with almost a century of observed U.S. presidential election data. Analyzing the social influence topography over this period reveals an abrupt phase transition from low to high levels of social contagion, and robust differences among regions. These results suggest that social contagion effects are becoming more instrumental in shaping large-scale collective political behavior, with implications on democratic electoral processes and policies.

## Introduction

The understanding of collective human dynamics in theoretical and real-life social systems gained increasing attention in recent decades [1–7]. At the core of these efforts are models that incorporate a collection of interconnected individuals that change their behavior based on micro-level processes of social influence exerted by their neighbors, but also based on individuals’ personal influences independent of social context. The macro-level characteristics of the system emerge as a product of the collective dynamics of these personal influences and micro-level social influence processes. The question of how to separate and measure the effect of social influence is therefore a major challenge for understanding collective human behavior. Although a variety of experimental [8–12] and observational [13–16] studies attempted to address this challenge, identifying the extent of social influence based on large-scale, macro-level observational data in the presence of unknown network structure remains largely unexplored. To close this gap, we present a simple and universal method for measuring social influence, taking the voter model of statistical physics as our basic dynamical system [2–4, 17–19]. We apply our model to understanding the collective dynamics of voting in US presidential elections—a topic at the core of collective political behavior.

The study of electoral behavior has attracted considerable attention by political scientists. Most studies of voting behavior in the United States and other democracies view vote choices as the result of several interrelated attitudinal and social factors [20]. Attitudinal factors that reflect short‐term fluctuations in partisan division of the vote include evaluations of the candidates' personal qualities and government performance, and orientations toward issues of public policy. Long-term factors, which persist beyond a particular election, include partisan loyalties [21–22], ideological orientations [23], and social characteristics such as race, religion, social class, and region [20]. Recent studies have also elucidated the role of social networks in spreading voting behavior [24]. Voters embedded in social networks of friends, family members, neighbors, and co-workers [12] influence each other in terms of voter turnout [12, 25–27] or support particular candidates [24, 28]. Social networks enable bounded rational voters to limit the cost of searching for political information [23] by relying on readily available information of their peers. These peer groups can also include “opinion leaders” who can considerably influence the behavior of voters in their network of contacts by being perceived as trustworthy and highly informed on political issues [21]. The opinion leaders (also known as “zealots,” “inflexible,” “stubborn” or “frozen” voters in the sociophysics literature, see [18–19, 29–38]) are individuals who hardly change their political preferences, and influence the voting behavior of uncommitted individuals. Opinion leaders often interpret media messages and pass them on to "opinion followers" [21]. Other sources of political information that were shown to influence citizen attitudes and voting behavior are the mass media [39–43] and a variety of organized efforts at political persuasion such as campaign persuasion [44].

Thus the picture that emerges from the modern history of social science academic voting research suggests that voters are embedded in interpersonal social networks that can increase the likelihood of voting contagion and behavior change via social imitation; but are also exposed to what we might call “external influences”—social forces, which are often consistently skewed in favor of one candidate over another [24], that affect voters. As mentioned above, these external influences include various individual prejudiced attitudes and orientations, party identification, individual’s upbringing, religion, ideology, campaign persuasion, and exposure to the mass media, such as television and newspapers. Since in this paper our focus is on understanding the dynamics of flexible voters who are free to change their voting behavior, without loss of generality, one can consider exposure to opinion leaders (including peers, journalists, or politicians), to be an external influence, despite sometimes being a peer influence effect. The reasoning for this is that opinion leaders are ideologically inflexible and unwavering candidate supporters, and thus convey a consistent partisan bias in favor of one candidate over another [24]. Collectively, the voter’s electoral decision can be explained in terms of peer effects (via social imitation) and partisan biases conveyed by competing external influences.

A pertinent question here is how to disentangle the effect of social contagion from that of exposure to external influences. This identification problem goes beyond voting. People hold opinions on a multitude of topics that inform alternative courses of action, from crime participation [45] and smoking [46] to riots and protests [47] and financial markets [31]. These opinions can be either the result of individual considerations or, when confronted with information that is difficult to acquire or process, influenced by the views of others through social interactions. In this paper we describe a general methodology for detecting behavioral contagion from large-scale observational data. We extend the basic voter model [2–4, 17–19] by taking into account the dynamic response of social networks to external influences. Our model focuses on two characterizations of voting behavior. The first is that of most studies of voting behavior, which consider vote choices to be driven, as outlined above, by various individual’s biases and other external pressures. The second—from complex systems science and recent observational and face-to-face studies—is that of internal self-reinforcing dynamics where voters’ opinions are changed under the influence of their peers. Incorporating both, we construct a universal representation of the largest scale system behavior when there is both external and interpersonal influence. The extended voter model is able to reproduce remarkably well statistical features and patterns of almost a century of county-level U.S. presidential election data. More importantly, our model presents a general framework for detecting social contagion from large-scale election return data, and can be applied more generally to many different systems that involve social contagion.

**Fig 1:**
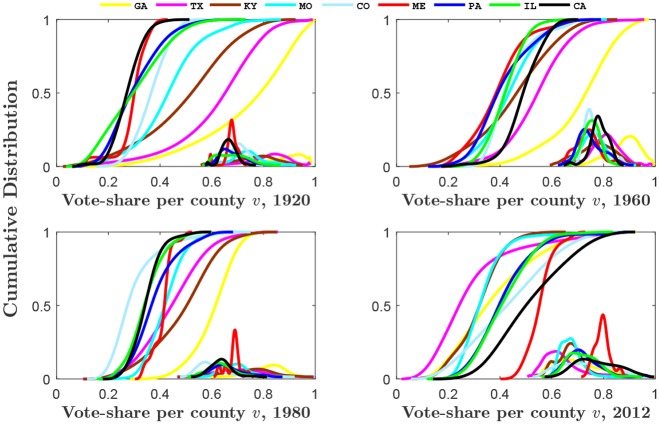
Observed county vote-share distributions, 1920–2012. The observed distributions of Democratic vote-share per county are presented for states with the greatest number of counties, for each of the nine census divisions established by the U.S. Census Bureau. Here plots are shown for various presidential elections from 1920 to 2012. The vote-share per county is measured as the percentage of the vote in the county received by the Democratic candidate. The figure shows the plot of the cumulative distributions. The insets show the corresponding probability density functions (for clarity the x-scale has been shifted to right and contracted). Curves are based on kernel estimation with Gaussian kernels.

Here, electoral votes cast for U.S. presidential candidates at the county level are analyzed, covering the period of 1920 through 2012. Counties are grouped by state, and the corresponding distributions of the fraction of votes (vote-share) in a county for the Democratic candidate in an election year are studied. Fig 1 shows the county vote-share distributions for various states and election years. The data indicates that there is a wide variation in the characteristics of voting behavior with no apparent pattern of voting dynamics in time or geographical space. Here we show that much of this observed variability of county vote-shares may be best explained by fluctuating peer influences across time and space. Although the study of collective voting behavior has recently been the focus of discussion in the context of identification and modelling of universal patterns of observed voting behavior [48–59], the mechanisms leading to such diverse spatiotemporal variation in voting patterns as shown in Fig 1 as well as other spatiotemporal patterns discussed in our paper (see section Empirical Results of US Presidential Elections: 1920 to 2012) are poorly understood. The model presented below provides a parsimonious quantitative framework that is capable to explain and reproduce, among others, the full range of empirical county vote-share distributions for all states and election years. Using the model, we develop an index of social influence that enables us to examine and reveal remarkably robust patterns of spatial and temporal variation in social influence over 92 years of US presidential elections. The statistical physics model presented in this paper is obviously limited in ignoring a lot of psychological and social factors influencing the decisions of individual voters. However, this limitation should not be perceived as an overly simplified assumption that overlooks human complexity. Indeed, as demonstrated by other models of social complexity [1–7], at times the details of a complex system do not matter if one wants to understand the large-scale behavior of the system. In this case, only the broad-brush features of the system are necessary to understand the complexity of human choices; in our case, the relative strength of external to peer influences are shown as a plausible explanation for observed voting behavior.

## Models of opinion dynamics

Models of opinion formation, which explore the dynamics of competing opinions taking into account the interactions among agents, have been extensively studied [1–7, 60–61]. In their most basic form, these models consist of voters, represented by nodes on a social network, having only two possible opinions, 0 or 1. Each voter may change her mind by using various interaction mechanisms, for example, randomly adopting the opinion of a connected neighbor (essentially a noisy majority-vote rule, see [3, 17, 58]), or by applying local majority rules [1, 3, 5]. The stochastic dynamics of these simple interaction models ultimately leads to a uniform state corresponding to the all-nodes-0 or all-nodes-1 states where all voters share the same political choices. Obviously, consensus states are not observed in real-world political elections, and thus the basic models cannot be plausibly considered as realistic models that are able to describe empirical voting data. Accordingly, more realistic models of opinion dynamics have been proposed that incorporate, among other features, social impact theory [60–62], opinion leaders and zealots [18–19, 29–38, 62–63], external influences and fields [2, 18–19, 64–70], individual’s biases [71–72], contrarians [73], individual’s own current opinion [74–75], word-of-mouth spreading [52], non-overlapping cliques [59], or noisy diffusive process [58]. Below we further elaborate on the themes of opinion leaders and zealots, external influences, and individual’s biases—themes that play an important role in our model, and that have been seen empirically by studies of electoral behavior (see Introduction).

Opinion leaders have often been modeled by considering the presence of biased voters who favor one opinion over the other, and that will not change their opinion (also known as zealots [29], inflexible [30], frozen [18–19, 31], stubborn [32–35], or committed voters [36–38]. The problem has been originally introduced and studied with a single zealot in regular lattices [29], and has been subsequently incorporated in models that use repeated local updates of random grouping of agents in the limit where the number of voters and zealots goes to infinity [30], and with arbitrary numbers of voters and zealots in fully connected networks where complete and exact results of the stochastic dynamics have been obtained [18–19]. Further studies that explore the use of zealots in the context of the voter model include [31–34, 36–38, 76].

The role of external influences (distinct from social imitation) in opinion formation has often been modeled as an external perturbation or modulation acting on all agents in the system, or by some external field or global coupling [3]. These perturbations could account for the effects of propaganda [65], fashion waves [66–67], or the mass media [68–70]; but are also driven by individual biases and prejudices [71–72], or level of political awareness [64]. More generally, these perturbations represent the dynamic response of a complex system to an external environment [18–19, 63]. As mentioned in the Introduction, this view is deeply aligned with empirically and theoretically grounded research by political scientists, which have uncovered external forces in the form of prejudiced attitudes and orientations of individuals, party identification, individual’s upbringing, religion, ideology, exposure to campaign persuasion and the mass media (such as television and newspapers), or partisan bias conveyed by opinion leaders.

While the above models show the important role of contagion spreading via social interactions in collective opinion dynamics, any further progress in understanding *real-world voting phenomena* needs to supplement and contrast these theoretical efforts with large sets of empirical voting data. Some effort has been done in this regards [52, 56, 58], particularly with the aim of explaining and reproducing the distribution of votes in bipartisan and proportional elections. Our paper is a contribution in this direction. Below we present an exactly solvable model of stochastic voter dynamics obtaining, among others, the stationary vote-share fluctuations across counties, which is in excellent agreement with almost a century of observed U.S. presidential election data at the state level, for every election year. The model is further validated by reproducing empirical temporal and spatial election patterns, as identified by social science academic voting research.

## The model

To model the dynamics of elections, we take the prototypical voter model [2–3, 17–19] as our basic dynamical system, and modify it to more closely reflect features of real-world political elections (see Introduction). We consider a network with *N* “free” nodes representing uncommitted voters [18–19, 29–38], and links between pairs of free nodes representing peer influences. We add to this network of uncommitted voters “fixed” nodes representing unwavering candidate supporters and opinion leaders that influence all other uncommitted voters, but are not themselves influenced by other nodes [18–19, 29–38]. The assumption that there exists a directed link from each uncommitted voter to each fixed node is motivated by the empirical fact that uncommitted voters are consistently exposed to partisan bias in favor of one of the candidates over another [20–22, 24, 39–44], conveyed by opinion leaders and other external sources (see detailed discussion below). The number of fixed nodes that are biased in favor of the first candidate (named ‘0’) is *N*^0^ and the number biased in favor of the second candidate (named ‘1’) is *N*^1^. Thus, we consider a network with *N* + *N*^0^ + *N*^1^ nodes. Each node has an internal state which can take only the values 0 and 1, representing whether the voter chooses the first or second candidate; or, for fixed nodes, whether the node is biased in favor of the first or second candidate. We assume a mean-field interaction model in which each uncommitted voter is equally likely to interact with another uncommitted voter [3, 17, 58, 77]. Accordingly, individuals update their contacts in a fully mixed fashion within the population, which implies a homogeneous random network for the uncommitted voters’ social ties. We assume that the *N free* voters change their internal state following the noisy majority-vote model [3, 17, 58]: At each time step a random free voter is selected and its state is updated with probability 1 − *p* by copying the state of one of its connected neighbors, chosen at random from all nodes; and with probability *p* the state remains the same. The *N*^0^ and *N*^1^ voters that are biased towards the first and second candidate, respectively, remain fixed in state 0 and state 1, respectively.

Our model’s assumptions and the noisy majority-vote update rule that we use [3, 17, 58] share important features with other variants of the majority rule principle. For example, the elegant majority rule proposed in [77]—see also the excellent review in [3]—assumes that all agents in the population can communicate with each other; forming, at each iteration, a random group of agents who take the majority opinion inside the group [3]. In this model, therefore, multiple individuals’ vote choices are updated simultaneously at each time step, at variance with our noisy majority-vote update rule where a single individual’s vote choice is updated at each time step [3]. The presence of inflexible agents with opposing views in this model [30, 35] leads to a solution, in the mean field limit [3, 77], that eventually settles into a fixed value of vote-share for one candidate, depending on the initial conditions. Our generalized voter model, on the other hand, does not necessarily settle into a fixed value. Instead, our main result shows that despite fluctuations of the voting dynamics, voter choices converge *in distribution*. Moreover, the long-run stationary distribution of vote-shares does not depend on the initial vote choices of uncommitted voters. This result, first reported in [18] within a fully solvable model, accounts for both the *finite* number of voters in a population and the numerous sources that convey *consistent partisan biases* to uncommitted voters. These properties and the foregoing model’s assumptions essentially create the kind of *characteristic vote-share fluctuations* across counties as recently observed in the sociophysics literature (e.g., [52, 56, 58, 78]) for various countries; and thus support the plausibility of the model and its capacity to describe real world voting phenomena. Of course, successful matching to a variety of spatiotemporal real-world election data is the ultimate test of *any* theory.

The parameters *N*^0^ and *N*^1^ of the fixed voters can be interpreted according to two viewpoints. We emphasize that both viewpoints are valid and useful: (1) Zealots and opinion leaders: As originally stated above, fixed voters can be viewed as unwavering candidate supporters and opinion leaders (peers, journalists, or politicians) that influence uncommitted voters, but are not themselves influenced by their neighbors' vote choices; (2) External factors: Alternatively, following our assumption in which each uncommitted voter is equally likely to interact with the fixed voters, the parameters *N*^0^ and *N*^1^ give the “effective strength” of the *consistent partisan bias* conveyed by the fixed voters in favor of one of the candidates (with effective strength *N*^0^) over another (with effective strength *N*^1^). As stressed in the Introduction, these consistent partisan biases by opinion leaders is merely one instance of a broad class of *consistent external factors* that influence the choices of uncommitted voters. These external factors include exposure to television, newspapers, or campaign persuasion. Recognizing that no voter is a “blank slate,” these external factors also include any prejudiced beliefs, party identification, individual’s upbringing, religion, or political ideology of uncommitted voters [20–22]. Mathematically, this broad interpretation is achieved (see Materials and methods) by analytically extending the parameters *N*^0^ and *N*^1^ to *non-integer values*; thus enabling modeling arbitrary strength of these external influences in favor of one of the candidates over another. According to this viewpoint, copying the state of a connected voter represents mutual influence among friends, neighbors, and family members via social imitation or via a consistent partisan bias acting on uncommitted voters (by opinion leaders or other external sources). External influences of opposite partisan biases do not cancel; instead larger *N*^0^ and *N*^1^ reflect increasing probability that consistent partisan biases determine the choices of uncommitted voters, independent of the voting choices of other uncommitted voters. Here we assume that there are many external sources of competing political information, and that over the election period in question the sources are persistent in their proportion of partisan biases regarding the two-major party candidates, though vary in the way they influence individual voters’ choices. Election years that are consistently biased towards the first (second) party’s candidate would be represented by *N*^0^ greater (smaller) than *N*^1^.

### The limiting stationary distribution of votes

We have previously proposed the above model as a widely applicable theory of collective behavior of complex systems [18–19, 31, 79–80], where the generalized voter model was solved exactly for a fully connected network. The fully connected network case was also shown to be equivalent (up to simple scaling) to a homogeneous random network (see Materials and methods). More specifically, at equilibrium, the probability of finding the network in the global state of *k* free voters in state 1 (i.e. voting for candidate 1) is given, independently of the initial state, as follows (see derivation in Materials and methods):

$$\rho \left(k\right)=\frac{\left(\begin{array}{c} N^{1}+k-1\\ k \end{array}\right)\left(\begin{array}{c} N+N^{0}-k-1\\ N-k \end{array}\right)}{\left(\begin{array}{c} N+N^{0}+N^{1}-1\\ N \end{array}\right)}.$$ (1)

where *N* is the number of free voters, *k* is the number of free voters is state 1 and $\left(\begin{array}{c} n\\ k \end{array}\right)$ are binomial coefficients. As mentioned above, analytically extending the parameters *N*^0^ and *N*^1^ to non-integer values enables to capture not only the case of zealots and opinion leaders but also the generalized effects of external factors (see Materials and methods). In this case, the solution in Eq 1 remains the same, with the difference that factorials must be replaced by gamma functions. Indeed, as we move around in the (*N*^0^, *N*^1^)-parameter space, the stationary distribution in Eq 1 exhibits strikingly different shapes. The different shapes of the stationary distributions depend on the magnitude of the external parameters, *N*^0^ and *N*^1^, compared to the extent of social imitation within the network of uncommitted voters, and the relative partisan bias of opinion leaders or other external influences (e.g., television and newspapers) toward the first or second candidate (i.e., *N*^0^ > *N*^1^ or vice versa). As shown in Materials and Methods, these distributions vary from skewed unimodal distributions with intermediate peaks or peaks at all nodes 1 or all nodes 0, to bimodal and uniform distributions. Interestingly, Eq 1 remains valid for other network topologies (including random, regular lattice, scale-free and small world networks) if *N*^0^ and *N*^1^ are re-scaled according to the degree distribution (see Materials and methods).

In this paper we are mostly interested in the fraction of voters (vote-share) that voted for a candidate rather than the actual number of voters. Thus, we define the vote-share for candidate 1 as the scaled variable *v* = *k*/*N*. The mean and variance of *v* can be computed from Eq 1 as follows

$$\mu_{v}=\frac{N^{1}}{N^{0}+N^{1}}$$ (2)

$$\sigma_{v}^{2}=\overset{\text{opinion leaders}/\text{external forces}}{\overset{︷}{\frac{\mu_{v}\left(1-\mu_{v}\right)}{N}}}\overset{\text{social imitation}/\text{peer influence}}{\overset{︷}{\left(\frac{N}{N^{0}+N^{1}+1}+\frac{N^{0}+N^{1}}{N^{0}+N^{1}+1}\right)}}$$ (3)

The variance of vote-shares in Eq 3 has an appealing interpretation. When peer influences (via social imitation) are very weak compared to external forces (*N*^0^, *N*^1^ → ∞), the variance of vote-shares becomes $\sigma_{v}^{2}=\mu_{v}\left(1-\mu_{v}\right)/N$. This is the variance of vote-shares that one would expect if all uncommitted voters are solely influenced, each with probability *μ*_*v*_, by the consistent partisan biases exerted by either opinion leaders with opposing views or other external forces (e.g., mass media), independent of the voting choices of other uncommitted voters. The second term on the right side of Eq 3, which is a decreasing nonlinear function of the external influence parameters, represents the effect of social imitation and peer influence within the network of uncommitted voters. This second term, which we call the “social influence index,” provides us with a method of detecting and isolating the effect of social imitation and social contagion. We use this index extensively in this paper to explore and understand how social influence changes across states and over almost a century of county-level U.S. presidential election years.

### Estimation of external influence from large scale voting data

The U.S. presidential election data are often collected at the level of counties. This data provides, among others, information on the vote-share in each county *i* (a single realization from an unobserved stationary vote-share distribution). Thus, in order to divulge the phenomenology of voting contagion in electoral voting behavior, we need to show how to estimate the external parameters of the generalized voter model from real data. The unknown external influence parameters *N*^0^ and *N*^1^ for any state in any election year can be estimated from a sample of observed vote-shares across counties as follows. Suppose a particular state has *n* counties, and let *v*_*i*_ be the fraction of voters in the *i*^th^ county that voted for candidate 1, and *N*_*i*_ be the total number of votes cast for all candidates in the county. We assume that all counties of a state are influenced by the same external parameters *N*^0^ and *N*^1^. Accordingly, the voting dynamics in the *i*^th^ county is governed by the generalized voter model, which applies to a subnetwork of *N*_*i*_ free nodes and *N*^0^ and *N*^1^ fixed nodes (note that each county has a different number of free nodes). We assume that the vote-share distribution (Eq 1) in each county is in equilibrium, and that the corresponding mean and variance are given by Eqs 2 and 3.

Using Eq 2, the expected value of the vote-share in county *i* does not depend on *i*, and is equal to *μ*_*i*_ = *μ* = *N*^1^/(*N*^0^ + *N*^1^). We thus estimate *μ*_*i*_ by simply taking the sample average $\hat{\mu }$ of vote-shares across all *n* counties. For the variance of the vote-share $\sigma_{i}^{2}$ in county *i*, a crude estimate based on the single observed vote-share data point *v*_*i*_ is provided by ${\left(v_{i}-\hat{\mu }\right)}^{2}$. Obviously, this estimate is imperfect and we define the residual between $\sigma_{i}^{2}$ and the estimate of $\sigma_{i}^{2}$

$$\varepsilon_{i}={\left(v_{i}-\hat{\mu }\right)}^{2}-\;\sigma_{i}^{2}$$ (4)

Using Eqs 3 and 4, we define a system of nonlinear estimation equations (one equation for each county) that relate ${\left(v_{i}-\hat{\mu }\right)}^{2}$, the estimate of $\sigma_{i}^{2}$, to the external parameters *N*^0^ and *N*^1^:

$${\left(v_{i}-\hat{\mu }\right)}^{2}=\;\frac{\mu \left(1-\mu \right)}{N_{i}}\left(\frac{N_{i}}{N^{0}+N^{1}+1}\;+\;\frac{N^{0}+N^{1}}{N^{0}+N^{1}+1}\right)\;+\;\epsilon_{i}\;\;\;\;i=1,\cdots ,n$$ (5)

The estimation procedure first estimates *μ* on the right hand side of Eq 5 by $\hat{\mu }$, and then select the sum of parameters *N*^0^ + *N*^1^ that minimizes the squared errors $\Sigma_{i=1}^{n}\varepsilon_{i}^{2}$ in Eq 5. The least squares estimate is given by

$${\hat{N}}^{0}+{\hat{N}}^{1}=\frac{n\hat{\mu }\left(1-\hat{\mu }\right)+\sum_{i}\frac{{\left(v_{i}-\hat{\mu }\right)}^{2}}{N_{i}}-\hat{\mu }\left(1-\hat{\mu }\right)\sum_{i}\frac{1}{N_{i}}-\sum_{i}{\left(v_{i}-\hat{\mu }\right)}^{2}}{-\hat{\mu }\left(1-\hat{\mu }\right)\sum_{i}\frac{1}{N_{i}}+\sum_{i}{\left(v_{i}-\hat{\mu }\right)}^{2}+\hat{\mu }\left(1-\hat{\mu }\right)\sum_{i}\frac{1}{N_{i}{}^{2}}-\sum_{i}\frac{{\left(v_{i}-\hat{\mu }\right)}^{2}}{N_{i}}}$$ (6)

Eq 6 and the condition $\hat{\mu }={\hat{N}}^{1}/\left({\hat{N}}^{0}+{\hat{N}}^{1}\right)$ fully determine the estimated external parameters. We can then use the estimate in Eq 6 to obtain the “social influence index" of the state as defined in Eq 3:

$$\frac{\bar{N}}{{\hat{N}}^{0}+{\hat{N}}^{1}+1}\;+\;\frac{{\hat{N}}^{0}+{\hat{N}}^{1}}{{\hat{N}}^{0}+{\hat{N}}^{1}+1}$$ (7)

where $\bar{N}=\Sigma_{i}N_{i}/n$ is the average number of voters per county. Eq 7 forms the basis for the statistical analysis of social imitation; for all states across U.S. presidential elections (see section Empirical Results of US Presidential Elections: 1920 to 2012).

### Derivation of the stationary vote-share distribution at the county level

For the U.S. presidential elections from 1920 to 2012, we empirically find that the external parameters ${\hat{N}}^{0},\;{\hat{N}}^{1}\gg 1$ for all states and across election years. Moreover, we notice that the total number of voters *N*_*i*_, in any county *i* for any given election year, is large. Thus, the voting dynamics in any county is applied to a network of voters with a very large number of free and fixed nodes. Driven by these facts, we find that in the limit *N*_*i*_ → ∞ the stationary distribution in Eq 1—characterizing the long run distribution of votes in the *i*^th^ county—is approximately a Gaussian distribution (see Materials and methods). More specifically, the asymptotic vote-share distribution in county *i* is given by a Gaussian $\rho \left(v_{i}\right)=1/\sqrt{2\pi \sigma_{i}^{2}}e^{\left[-{\left(v_{i}-\mu_{i}\right)}^{2}/2\sigma_{i}^{2}\right]}$ with mean *μ*_*i*_ = *μ* = *N*^1^⁄(*N*^0^+*N*^1^) and variance $\;\sigma_{i}^{2}=\mu \left(1-\mu \right)\left(\frac{1}{N^{0}+N^{1}}+\frac{1}{N_{i}}\right)$. We stress that this predicted Gaussian vote-share distribution (and its characteristic mean and variance) *at the county level* is not assumed from the outset but turns out to be the consequence of basic principles of voting behavior and the generalized voter model. We next derived the stationary vote share fluctuations *across counties*.

### Derivation of the stationary vote-share distribution across counties

While the stationary vote share distribution at the county level is not observed (but predicted to be Gaussian), the availability of large sets of empirical voting data enables us to obtain, for each state in every election year, the probability distribution of observed vote-shares across all *n* counties in the state (see Fig 1). As mentioned above, this candidate vote share distribution has been the focus of recent attention.

A plausible model for the stationary vote-share distribution across counties is to describe it as a Gaussian scale mixture [81] with *n* different components (representing the *n* counties in the state), each distributed as a normal distribution with the same mean *μ* and different variances $\sigma_{i}^{2}$, as specified above. Let *v* denote the random variable corresponding to this Gaussian mixture (this is called the “vote-share per county” in Fig 1). This Gaussian mixture is a unimodal distribution with mode at *μ*, skewness value *β*_1_ = 0, kutosis value $\beta_{2}=3\Sigma_{i=1}^{n}\frac{1}{n}\sigma_{i}^{4}/{\left(\Sigma_{i=1}^{n}\frac{1}{n}\sigma_{i}^{2}\right)}^{2}$, and variance $\sigma_{v}^{2}=\Sigma_{i=1}^{n}\frac{1}{n}\sigma_{i}^{2}$ Using the Pearson system, the Gaussian scale mixture can be shown to be approximately a *t*-distribution [82]. More specifically, Let $c_{0}=\;2\sigma_{v}^{2}\beta_{2}/\left(5\beta_{2}-9\right)$ and *c*_2_ = (*β*_2_ − 3)/(5*β*_2_ − 9) be the Pearson coefficients corresponding to the Gaussian mixture, and let $\alpha =\sqrt{\left(1-c_{2}\right)/c_{0}}$, and *m* = (1 − *c*_2_)/*c*_2_. Then, the scaled and shifted random variable *α*(*v* − *μ*) is approximately distributed as a Student's *t*-distribution with *m* degrees of freedom [82]. Notice that the parameters *μ*, *α*, and *m* of this Student's *t*-distribution can be completely specified once the external parameters *N*^0^, *N*^0^ are estimated (as was shown above), and the number of counties in the state *n*, and total number of votes *N*_*i*_ in each county are given (these data are publicly available in many countries).

The above key result implies that the scaled and shifted vote-shares across counties can be described by a Student's *t*-distribution with *m* degrees of freedom. Finally, we empirically find for our comprehensive U.S. presidential election data that the number of degrees of freedom *m* ≫ 100 for all states in every election year. In this case, the Student's *t*-distribution with *m* degrees of freedom approaches the normal distribution; and thus the distribution of the scaled and shifted vote-shares across counties is predicted to match nicely the standard normal distribution. We emphasize that this predicted Gaussian vote-share distribution across counties is derived from first principles and does not involve any a priori assumption about the vote-share distribution. Successful matching to election data will be a corroboration of this theory.

## Empirical results of US presidential elections: 1920 to 2012

### Analyzing the county vote-share probability distributions

**Fig 2:**
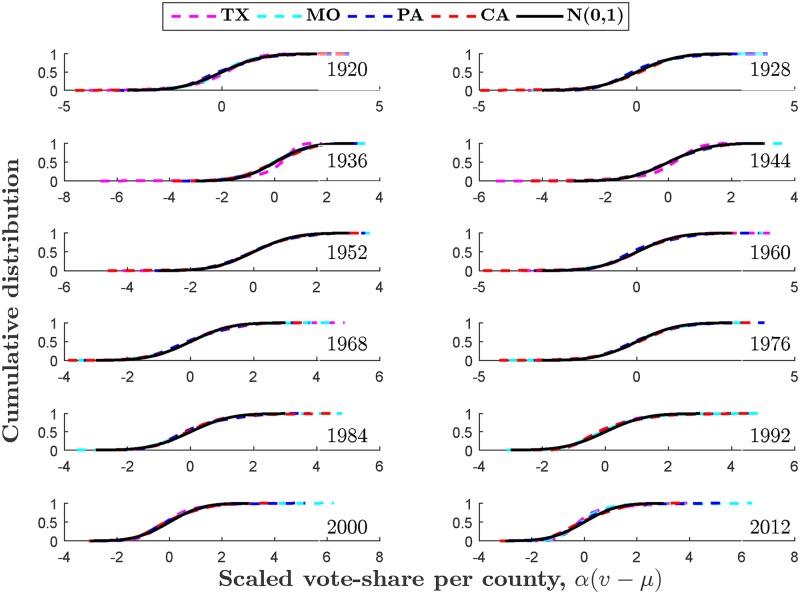
Scaled vote-share distributions and predicted curves, 1920–2012. Without loss of generality, curves are presented for states with the greatest number of counties in each of the four census regions—Northeast, Midwest, South, and West—of the U.S. The figure shows the plot of the cumulative distributions. Observed values (dashed lines) are based on kernel estimation with Gaussian kernels. Solid lines are Gaussian distributions with mean 0 and variance 1. The scaled vote-shares are calculated from the estimated external influence parameters *N*^0^ and *N*^1^. The goodness of fit of the Gaussian relative to the empirically observed county vote-share distributions was determined by using a Kolmogorov-Smirnov test. The fit of the model is excellent—the test fails to reject the normality null hypothesis at the 5% significance level, for 95% of all states in every election year; and at the 1% significance level, for 98% of all states in every election year.

Our analysis is based on US presidential election data from 1920 to 2012 [83]. States with less than 10 counties (i.e., Connecticut, Delaware, Hawaii, and Rhode Island) and Washington D.C. were excluded from analysis. For each state, in every election year, the data includes information on the number of counties *n* for which vote-share data was available, the vote-share *v*_*i*_ in county *i*, and the total number of votes cast for all candidates in county *i*, *N*_*i*_. The external influence parameters *N*^0^ and *N*^1^, and the distribution parameters *α* and *m* were estimated for all states and every election year. Using these parameters, we constructed the probability distribution of the scaled and shifted vote-share quantity *α*(*v*_*i*_ − *μ*), and compared it with the predicted normal distribution. Fig 2 shows that this theoretical prediction fits remarkably well for most states and election years, representing almost a century of county-level U.S. presidential election data, and is consistent with observations in other countries [78].

### Analyzing the evolution of social influence

**Fig 3:**
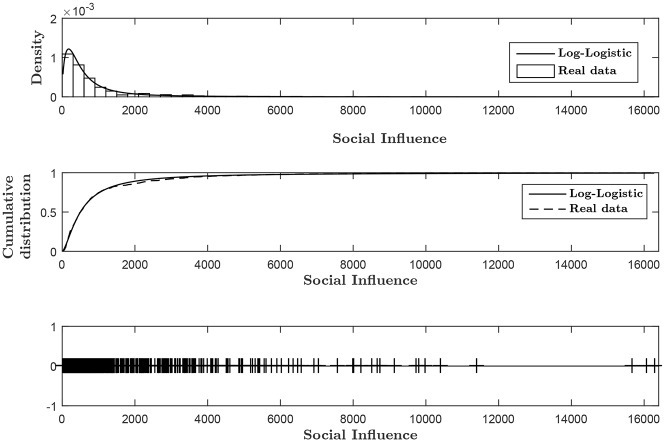
Distributions of social influence and best-fit curves, 1920–2012. The upper and middle panels of Fig 3 show the histogram and cumulative distribution of the Social Influence index (using Eq 7), aggregated over all states and election years. The lower panel shows the spread of the Social Influence index. The broad distributions are best fitted by the log-logistic distribution, often used for analyzing skewed data. The goodness of fit of the log-logistic distribution was determined by using a Kolmogorov-Smirnov test. The fit of the model is very good (p = 0.16).

We can use the index of social influence defined in Eq 7 to explore the level of social interactions across states and election years. We first examine the distribution of the social influence index, aggregated over all states and election years. The histogram in the upper panel of Fig 3 shows a right-skewed distribution. This means that while the bulk of the distribution occurs for small values of social contagion, the electorate in US presidential elections is at times highly volatile and subject to wide swings of social contagion effects higher than the typical value. This is reflected by the highly right-skewed tail of the histogram. Here we find that the log-logistic provides a slightly better fit relative to the log-normal distribution. The log-logistic is a heavy-tailed distribution similar in shape to the log-normal distribution, but with heavier tails [84].

**Fig 4:**
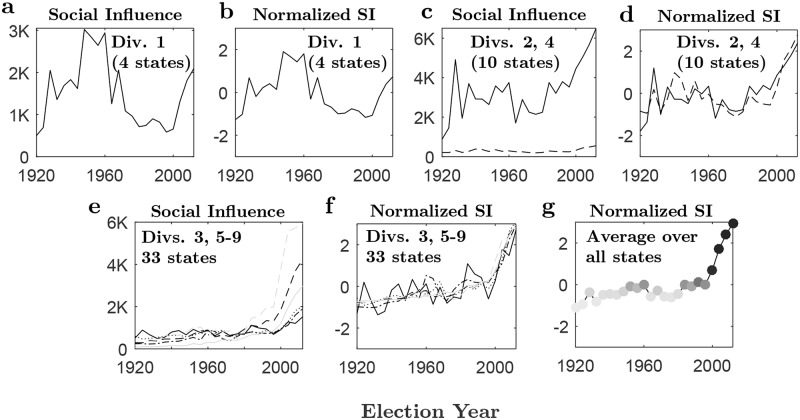
Evolution of social influence, 1920–2012. Time series of social influence averaged over states, and their normalized versions (see text), are shown for each of the nine U.S. census divisions: **a-b)** Division 1, New England—Maine, Massachusetts, New Hampshire, and Vermont; **c-d)** Division 2, Middle Atlantic (Solid line): New Jersey, New York and Pennsylvania. Division 4, West North Central (Dashed line): Iowa, Kansas, Minnesota, Missouri, Nebraska, North Dakota and South Dakota. **e-f)** Division 3, East North Central (Solid line): Illinois, Indiana, Michigan, Ohio and Wisconsin. Division 5, South Atlantic (Dashed line): Florida, Georgia, Maryland, North Carolina, South Carolina, Virginia and West Virginia. Division 6, East South Central (dotted line): Alabama, Kentucky, Mississippi and Tennessee. Division 7, West South Central (Dash-dot line): Arkansas, Louisiana, Oklahoma and Texas. Division 8, Mountain (Gray solid line): Arizona, Colorado, Idaho, Montana, Nevada, New Mexico, Utah and Wyoming. Division 9, Pacific: Alaska, California, Oregon and Washington (Gray dashed line). The nine U.S. census divisions are clustered according to the correlation between their respective normalized social influence profiles. The corresponding average pairwise correlations are 0.824 and 0.909 for the clusters in Fig 4c-d and Fig 4e-f, respectively. Panel 4g shows the time series of normalized social influence, averaged over all U.S. states. The *Z*–scores are mapped to colors from white (z = −1.1, below the mean) to black (*z* = 2.9, above the mean). A clear pattern of high social influence (positive or near-zero *Z*–scores) follows a period (1920–1980) of low social influence (negative *Z*–scores). The 1984 break date—separating low from high levels of social contagion—is identified by the Mann-Whitney U-test, which is applied for different potential breaks within the range 1920–2012 (see S1 Fig).

To analyze the electoral dynamics, we examine the spatial and temporal variation in social influence from 1920 to 2012. First, we examine the evolution of social influence over time. Fig 4 shows the time series of the average social influence for each of the nine U.S. census divisions (panels a-f) along with the time series of (normalized) social influence averaged over all U.S. states (panel g). To enable the comparison of the various time series, all data are normalized *Z*–scores. Specifically, for each individual time series we express the social influence in terms of standard deviation from their mean, calculated from 1920 to 2012. We use hierarchical clustering to identify clusters of U.S. census divisions with the highest within-cluster time-series correlation and the greatest between-cluster time-series variability. The result from the hierarchical clustering suggests three clusters: two main clusters (arranged in panels d and f) with within-cluster average correlations of 0.824 and 0.909, and a relatively high between-cluster average correlation of 0.775; and a singleton cluster (New England) with a relatively low between-cluster average correlation of 0.1071.

Remarkably, we find that despite variations in social influence across states and divisions, the normalized time series pertaining to the overwhelming number of states (with the exception of the three-state region of New England analyzed here) collapse on a very similar curve. Indeed, as can be seen, the normalized curves in panels d and f show a very similar pattern, which is also similar to the observed temporal pattern of social influence when averaged over all states (Fig 4g). That is, the pattern in Fig 4g shows a monotonic upward trend, which means that social influence increases through time (Mann-Kendall test, p < 0.001). Moreover, the period of 1984–2012 displays much higher levels of social influence when compared with the period of 1920–1980, which displays lower levels of social influence (Mann-Whitney U-test, p < 0.001, see S1 Fig).

New England is an apparent exception to this pattern (Fig 4a and 4b). However, this exception may be explained by the historical events and our model. One of the most unique characteristics that makes New England, as a political region in America, different from other regions is its town meeting form of government—a local institution that did not spread to other states [85]. The town meeting is the legislative assembly of a town in which qualified voters make laws in face-to-face communal decision making [85]. Town meetings defined New England’s politics until the middle decades of the 20^th^ century. This was changed in 1962 with the Supreme Court’s “one person, one vote” decisions, which resulted in shifting power dynamic away from most small towns that practiced town meetings, face-to-face interactions, to cities that adopted representative politics [85]. Thus the relative high levels of social interaction observed in New England prior to the 1960 election (see Fig 4b)—contrary to the patterns observed in other regions—correspond to the period in which town meetings—a powerful platform of social influence via face-to-face, communal, decision making—had wide legislative powers. This was followed by a sharp decline in relative social influence (see Fig 4b) after the Supreme Court’s “one person, one vote” decision, which had the effect of shifting the power from face-to-face communication and social interaction to representative politics. This political transition changed not only the relative level of social interactions—and thus the variability of the vote-share distributions—but also impacted the partisan bias—hence the mean of the vote-share distributions—towards the Democrats [85].

### Analyzing the correlation trends of social influence

**Fig 5:**
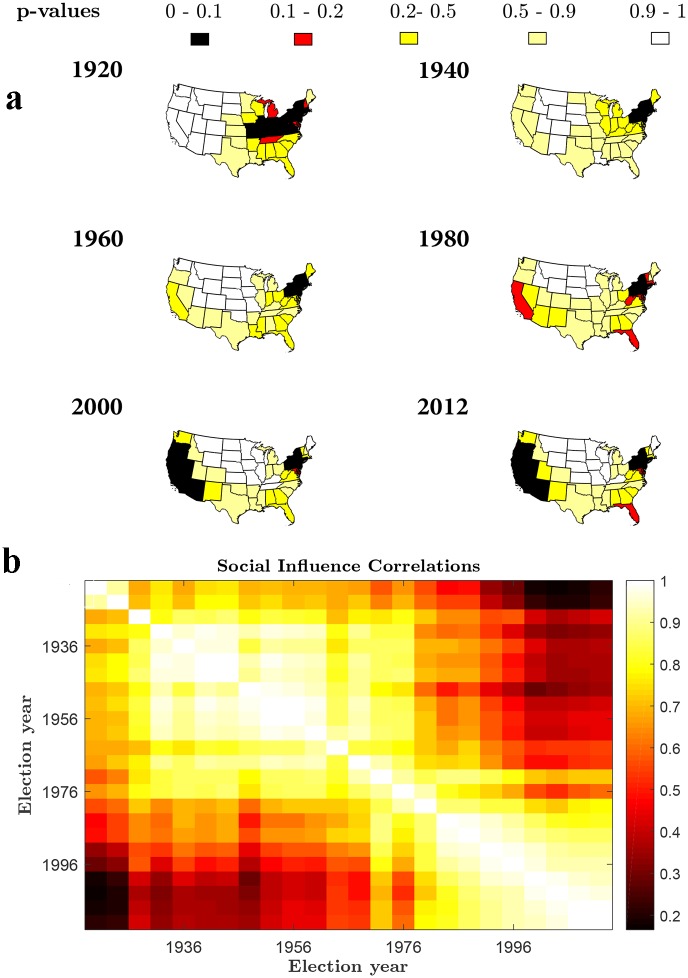
Spatial variation of social influence and its change over time, 1920–2012. **a)** Hot spot analysis of social influence for sample maps of US presidential elections (see S2–S25 Figs and S1 Movie for a complete analysis). The colored areas reflect the significance (p-value) of local concentration of social influence for each state. The p-values for each state are derived from a random permutation test of local clustering using the Getis-Ord Local ${\text{G}}_{i}^{*}$ statistic. This analysis was performed with a contiguity spatial weight matrix that indicates whether states share a boundary or not. The variable of concern is the social influence index calculated using Eq 7 in the main text. Low p-values (p-value≤0.1) indicate statistically significant high levels of social influence at a state and its surrounding neighbors (hot spots). High p-values (p-value≥0.9) indicate statistically significant low levels of social influence at a state and its surrounding neighbors (cold spots). **b)** Heatmap of the correlation coefficients between pairs of election years characterized by their state-level social influence profiles.

As a further support for the usefulness and consistency of our model, we examine how the spatial variation of social influence across states changes over time. We can characterize each election as a vector of state-level indices of social influence (using Eq 7), and measure the similarity between each pair of elections by the corresponding correlation coefficient (Fig 5b). This type of analysis, combined with the findings in Fig 4, reveals intriguing patterns that go beyond short‐term fluctuations in partisan division of the vote. Hierarchical clustering of the elections by the social influence correlation distance shows several marked clusters of highly similar election years (see Fig 4b): 1932–1972, 1984–2012, and three smaller clusters 1920–1924, 1928, and 1976–1980. Remarkably, these clusters of social interactions correspond nicely with the partitioning of American history into distinct party systems [86].

There have been six party system periods in American history, separated by relatively significant change in party loyalties [86–90]. Clustering analysis reveals that during 1932–1972, external forces (in the form of attitudes, orientations, party identification, individual’s upbringing, religion, or ideology) are strong compared to social/peer influences, and indicate a stable long-term electorate phase. This result is plausibly supported by the historical account. The stock market crash of 1929 and the ensuing depression signaled the realignment of the fifth party system from a Republican to Democratic majority with the election of 1932 and the New Deal coalition [86, 88]. The change was also influenced by demographic changes of rising American electorate of African Americans, blue collar workers, Catholics and urban ethnics, and a shrinking Republican base of white Protestants, small town residents, farmers, and middle class businessmen [88]. The distinction between external and social influence stands despite some fluctuations in Republican vs. Democratic selections.

The cluster 1976–1980 identified by our clustering analysis (see also Fig 5b) suggests that the elections of 1976 and 1980 formed a transition period to the post-New Deal era of weakened partisanship among the voters [88]. This transition period corresponds to the Watergate scandal and Nixon's resignation in 1974, and Democrat Jimmy Carter’s victory in the 1976 presidential election.

Whereas the previous fifth party system was characterized by strong party loyalties and partisan attachments, the sixth party system (overlapping with the 1984–2012 cluster in Fig 5b) is characterized by electoral dealignment—the weakening of party loyalties among voters [88, 90–91], reduced political involvement [92], and the critical role of voters’ personal social interaction networks in determining vote choices [24]. As partisanship declined and more voters became independents [86], inter-election vote swings increased [88]. Moreover, the external influence of television and newspaper declined as the media were considerably less likely to be sources of partisan-biased information [24, 88, 93]. This led to a period of strong competition where neither Democrats nor Republicans created a true majority party, resulting in alternating control of the presidency, split-ticket voting, and divided government. These trends seem to be consistent with our model, which shows higher levels of social contagion for the 1984–2012 period (Fig 4g), relative to the 1920–1980 period, combined with the long-term stability of social influence patterns indicated by the high levels of association between the 1984–2012 elections (Fig 5b). The high levels of social interactions observed in the 1984–2012 period (Fig 4g) account for an increasing volatility and variability of the vote-share distributions (via Eq 3). This is seen in the historical account: The Republicans won the presidency with the victories of Ronald Reagan and George H. W. Bush in 1980, 1984, and 1988, and regained control of the Senate from 1981 to 1987 for the first time in almost 30 years. The Democrats regained control of the presidency with Bill Clinton in 1992 and 1996 whereas the Republicans won control of the Congress from 1994 to 2006 for the first time in 40 years. In 2000, Republican George W. Bush defeated Democratic Al Gore in the closest election in modern U.S. history. Although Bush won reelection in 2004, Democrats won control of Congress in 2006, and Democrat Barack Obama was elected in 2008. Although it would seem that Obama's victories in the 2008 and 2012 suggest a critical realignment of the party system, the Republicans regained control of the House in 2010 by their biggest landslide since 1946, and control of Congress in 2014, with the largest Republican majority in the House since 1928.

### Mapping the geography of social influence

S2 Movie in Supplementary Material shows maps (excluding Alaska and Hawaii), color-coded by levels of social influence, for all election years. In order to better characterize the spatial patterns of social influence observed in S2 Movie, we apply a variety of spatial statistical data analysis methods. First, we utilized a random permutation test of spatial autocorrelation using the Moran’s *I* statistic [94, 95]. The random permutation tests suggest (see S1 Table) the presence of significant positive spatial correlation, for all election years, between states’ own levels of social influence and the levels of their neighbors as indicated by the level of significance (p-value) shown in the third column of S1 Table. This analysis was performed with a contiguity spatial weight matrix (row normalized) that indicates whether states share a boundary or not. While the Moran’s *I* statistic indicates that the spatial distribution of high and/or low values is more spatially clustered than would be expected if underlying processes were random, it does not identify unexpected spatial spikes of high or low social influence values. We thus applied random permutation tests of spatial clustering using the Getis-Ord General G* statistic [96–97]. The tests indicate (see S2 Table) that social influence is significantly concentrated in space as shown by the significance levels (p-value) in the third column of S2 Table. That is, for all election years, the observed Getis-Ord General G* is larger than the expected General G*, indicating that the spatial distribution of high social influence values is more spatially clustered than would be expected if underlying spatial processes were truly random.

In order to identify where high or low values of social influence cluster spatially, we further applied a random permutation test of local clustering using the Getis-Ord Local ${\text{G}}_{i}^{*}$ statistic [96–97]. Low p-values of the random permutation test indicate statistically significant high levels of social influence at a state and its surrounding neighbors (hot spots). High p-values indicate statistically significant low levels of social influence at a state and its surrounding neighbors (cold spots). This analysis was performed with a contiguity spatial weight matrix that indicates whether states share a boundary or not. The corresponding maps of hot spot analysis, for all election years, are presented in S2–S25 Figs and S1 Movie. A sample of these maps of social influence clusters is presented in Fig 5a. The colored areas in Fig 5a reflect the significance (p-value) of local concentration of social influence for each state, derived from the random permutation test of local clustering using the Getis-Ord Local ${\text{G}}_{i}^{*}$ statistic. The maps shown in Fig 5a (see S2–S25 Figs and S1 Movie for a complete analysis) enable to identify unusual geographical concentrations of high or low values (i.e., hot or cold spots) of social influence across the United States, for each election year. More specifically, the hotspot analysis of US presidential elections from 1920 to 2012 reveals a distinctive geographical cluster of states with statistically significant low levels of social influence (cold spots). This cluster is comprised of states mainly in the Great Plains and West North Central regions (including, for example, Montana, Wyoming, North Dakota, South Dakota, Nebraska, Kansas and Oklahoma). Contrastingly, states predominantly in the Middle Atlantic region (New Jersey, Pennsylvania, and New York)—for all election years—and states in the Pacific region (California, and Oregon) and the Southwest (Arizona and Nevada)—from 1988 to 2012—display high values of social influence (hot spots).

It would be interesting to speculate on the political, economic, social, and psychological factors that drive geographic variation in voting contagion. Research in the geographical and psychological sciences, which examines the geographical distribution of political, economic, social, and personality traits within the United States [98–103], suggests that the Great Plains and West North Central region is characterized by individuals that are typified by conservative social values, low openness and resistance to change, and preference of familiarity over novelty. This region comprises states with comparatively small minority populations [101], is less affluent, has fewer highly educated residents, is less innovative compared with other regions, and tends to be politically conservative and religious [100]. Individual in this region choose to settle near family and friends and maintain intimate social relationships with them, but also tend to display low levels of social tolerance and acceptance for people who are from different cultures, unconventional, or live alternative lifestyles [100]. Altogether, the above characteristics indicate a region where voters' choices are plausibly based upon strong ideology, party identification, orientations and attitudes rooted in religion and traditional social values, and reinforced by face‐to‐face interactions with like‐minded family members and friends. We therefore expect our model to generate a social influence index (see S2 Movie for maps of raw social influence values instead of the Getis-Ord Local ${\text{G}}_{i}^{*}$ statistic) that reflects external forces (e.g., in the form of party identification or ideology), which are strong compared to peer influences.

Unlike the very low openness and conservative social values typical for the Great Plains and West North Central region, states along the Middle Atlantic and Southwest region are marked by moderately to very high openness, is wealthy, educated, culturally and ethnically diverse, and economically innovative [100, 102]. This region appears to be politically liberal, and has fewer mainline Protestants [100]. Residents of this region also appear to be tolerant and accepting of social and cultural differences [100]. Considering the social diversity, tolerance, openness, and open-mindedness in this region, it is plausible that people’s orientations and attitudes are influenced by the attitudes of others [100]. This is consistent with our model, which shows high levels of social influence index (see maps of raw social influence values in S2 Movie) that indicate peer influences that are strong compared to external forces in the form of attitudes or ideology. Although further research is needed to uncover the factors affecting social influence, it is plausible that economic, social, and psychological factors, as discussed above, can explain the geographical variability of social influence.

## Discussion

Many complex systems can be viewed as comprising of numerous interconnected units each of which independently responds to external forces, but is also affected by internal forces exerted by the states of its connected units. In such systems, the stationary distribution of the states of the units may change in characteristic ways depending on the strength of external influences relative to internal influences [18–19, 31, 79–80]. Therefore, a key question is how to disentangle the effect of internal influences from that of exposure to external influences, given observational data about the phenomena we are trying to explain. This identification problem is important not only to the biological and physical sciences (e.g., ecosystems, see [104]), but also in the social sciences where the importance of social interactions in forming opinions and decisions has been emphasized [12, 45–46, 105]. The U.S. presidential elections are a case in point. In such situations, voters’ candidate choices are affected by many sources that convey *consistent partisan biases* skewed in favor of one candidate over another. These sources are numerous and include exposure to television, newspapers, campaign persuasion, or opinion leaders (including peers, journalists, or politicians); but also include various individual prejudiced attitudes and orientations, party identification, individual’s upbringing, religion, or ideology (no voter is a ‘blank slate’). Uncommitted voters are also affected by the choices of other uncommitted voters in their own personal networks, via social imitation mechanisms. All of these empirical facts are deeply rooted in the extensive study of electoral behavior by social and political scientists (see Introduction) as well as studies of opinion dynamics in the sociophysics literature (see Models of Opinion Dynamics). The vote-share fluctuations across counties, and other spatiotemporal voting patterns, thus depend on the relative magnitude of the persistent partisan biases for one candidate over another.

Individual voters are influenced by a variety of psychological and social factors, but taking them all into account would be not only impossible but also unnecessary for understanding the large-scale behavior of the system. This large-scale behavior can still be captured by introducing a few key parameters, as we have demonstrated in this paper. We presented a general methodology for quantifying the degree of social imitation and peer influence on the basis of given observational data. The methodology is based on an extended version of the voter model [18–19] that takes into account the effect of external forces, and is applied to a comprehensive data of US presidential elections from 1920 to 2012. An essential element in the model is social interaction between individual voters. The model includes two parameters that reflect the bias in favor of one of two candidates. These tunable parameters represent unwavering candidate supporters (zealots or opinion leaders) that convey a consistent partisan bias in favor of one candidate over another; or, as discussed above, alternatively can be interpreted as external factors that influence uncommitted voters’ choices. In addition to these external factors, voters are also influenced by the behavior of others via social imitation.

Our model is validated in several ways. First, we derive the theoretical probability distribution of the vote-share per county, and find a remarkable fit between the theoretical result and the empirically observed county vote-share distributions. Our theoretical result is also consistent with observations in other countries [78]. To our knowledge this is the first study that provides an analytical expression of the stationary vote-share distribution across counties. Second, we examined the temporal dynamics of social influence by calculating the social influence index for each state and each election year. Our analysis reveals a distinct pattern of increasing social influence over 92 years (1920–2012) of US presidential elections. The 1984 election year represents the phase transition point from low (1920–1984) to high (1984–2012) levels of social contagion. The increasing levels of social influence at presidential elections suggest, in turn, the decline of bias induced by external forces (e.g., partisanship among voters), and an increasing of independence in voting behavior. Third, we examined how the geographic variation across states in social influence changes over time. This spatiotemporal analysis enables our model to reproduce two stable long-term periods of election years corresponding to two successive long-term periods of low and high levels of social contagion, in alignment with the 1984 phase transition finding. This suggests a new data-driven, large-scale systems approach of characterizing abrupt transitions of political events, which is based on critical realignment in the patterns of social contagion. Finally, we use the model to map the social contagion geography of the United States. Results from spatial analysis reveal robust differences among regions of the United States in terms of their social influence index. In particular, we identify two regions of ‘hot’ and ‘cold’ spots of social influence, each comprising states that are geographically close. We provided some evidence that statewide variation in social contagion may be linked to psychological, social, and economic factors.

More broadly the results suggest the growing role of social influence, contagion, and ‘herd-following’ in shaping peoples’ behaviors, tastes, and actions in a variety of real-life situations. Social influence and contagion will likely become increasingly evident as our society becomes more interconnected through the information superhighway and transport infrastructure networks. If we want to truly understand macro-level collective behavior in human systems—and perhaps devise ways by which human society can increase its collective wisdom—it will be important to develop practical and effective methods for measuring and monitoring the extent of social influence.

## Materials and methods

### Dynamic network model of voting

Consider a network representing a county with *N* nonpartisan voters (variable nodes) taking only the values of 0 or 1, representing support for candidate 0 or 1, respectively (e.g., Republican or Democrat). In addition, there are *N*^0^ and *N*^1^ partisan voters (frozen nodes) in state 0 and 1, respectively. At each time step, a variable node is selected at random; with probability 1 − *p* the node copies the state of one of its connected neighbors, and with probability *p* the state remains unchanged. The partisan nodes can also be interpreted as external perturbations, representing a variety of factors that influence voters’ attitudes towards one of the two candidates (e.g., mass media, party identification, individual’s upbringing, religion, or ideology). Analytically extending *N*^0^ and *N*^1^ to be real numbers enables modeling arbitrary strengths of external perturbations.

For a fully connected network the behavior of the system can be solved exactly as follows. The nodes are indistinguishable and the state of the network is fully specified by the number of nodes with internal state 1. Therefore, there are only *N +* 1 distinguishable global states, which we denote *S*_*k*_, *k* = 0,1,⋯,*N*. The state *S*_*k*_ has *k* variable nodes in state 1 and *N* − *k* variable nodes in state 0. If *P*_*t*_ (*k*) is the probability of finding the network in state *S*_*k*_ at time *t*, then *P*_*t*+1_(*k*) can depend only on *P*_*t*_(*k*), *P*_*t*_(*k* + 1) and *P*_*t*_(*k* − 1). The probabilities *P*_*t*_(*k*) define a vector of *N* + 1 components **P**_*t*_. The dynamics is described by the equation

$$\begin{array}{ll} P_{t+1}\left(k\right) & =P_{t}\left(k\right)\left\{p+\frac{\left(1-p\right)}{N\left(N+N^{0}+N^{1}-1\right)}\left[k\left(k+N^{1}-1\right)+\left(N-k\right)\left(N+N^{0}-k-1\right)\right]\right\}\\ & +P_{t}\left(k-1\right)\frac{\left(1-p\right)}{N\left(N+N^{0}+N^{1}-1\right)}\left(k+N^{1}-1\right)\left(N-k+1\right)\\ & +P_{t}\left(k+1\right)\frac{\left(1-p\right)}{N\left(N+N^{0}+N^{1}-1\right)}\left(k+1\right)\left(N+N^{0}-k-1\right). \end{array}$$

The term inside the first brackets gives the probability that the state *S*_*k*_ does not change in that time step and is divided into two contributions: the probability *p* that the node does not change plus the probability 1 − *p* that the node does change but copies another node in the same state. In the latter case, the state of the node is 1 with probability *k* / *N*, and it may copy a different node in the same state with probability (*k* − 1 + *N*^1^)/(*N* + *N*^0^ + *N*^1^ − 1). Also, if the state of the selected node is 0, which has probability (*N* − *k*)/*N*, it may copy another node in state 0 with probability (*N* − *k* − 1 + *N*^0^)/(*N* + *N*^0^ + *N*^1^ − 1). The other terms are obtained similarly.

In terms of **P**_*t*_, the dynamics is described by the equation

$$P_{t+1}=TP_{t}\equiv \left(1-\;\frac{\left(1-p\right)}{N\left(N+N^{0}+N^{1}-1\right)}A\right)P_{t}$$ (8)

where the time evolution matrix **T**, and also the auxiliary matrix **A**, is tri-diagonal. The non-zero elements of **A** are independent of *p* and are given by

$$\begin{array}{lll} A_{k,k} & = & 2k\left(N-k\right)+N^{1}\left(N-k\right)+N^{0}k\\ A_{k,k+1} & = & -\left(k+1\right)\left(N+N^{0}-k-1\right)\\ A_{k,k-1} & = & -\left(N-k+1\right)\left(N^{1}+k-1\right) \end{array}$$ (9)

The transition probability from state *S*_*M*_ to *S*_*L*_ after a time *t* can be written as

$$P\left(L,\;t;M,\;0\right)=\overset{N}{\underset{r=0}{\sum }}b_{rM}a_{rL}\lambda_{r}^{t}$$ (10)

where *a*_*rL*_ and *b*_*rM*_ are the components of the right and left *r*-th eigenvectors of the evolution matrix, **a**_*r*_ and **b**_*r*_. Thus, the dynamical problem has been reduced to finding the right and left eigenvectors and eigenvalues of the time evolution matrix **T**.

The eigenvalues *λ*_r_ of **T** are given by

$$\lambda_{r}=1-\frac{\left(1-p\right)}{N\left(N+N^{0}+N^{1}-1\right)}r\left(r-1+N^{0}+N^{1}\right)$$ (11)

and satisfy 0 ≤ *p* ≤ *λ*_*r*_ ≤ 1. The equation for *P*(*L*,*t*; *M*, 0) shows that the asymptotic behavior of the network is determined only by the right and left eigenvectors with unit eigenvalue, i.e., by the eigenvector corresponding to *λ*_0_ = 1. The coefficients of the corresponding (unnormalized) left eigenvector are simply *b*_0*k*_ = 1. The coefficients *a*_0*k*_ of the right eigenvector are obtained using a generating function technique and an associated nonlinear second order differential equation [18–19]. The coefficients are then given by the Taylor expansion of the hypergeometric function *F*(−*N*, *N*^1^, 1 − *N* − *N*^0^, *x*) ≡ ∑_*k*_ *a*_0*k*_*x*^*k*^. After normalization, these coefficients give the stationary distribution

$$\rho \left(k\right)=\frac{\left(\begin{array}{c} N^{1}+k-1\\ k \end{array}\right)\left(\begin{array}{c} N+N^{0}-k-1\\ N-k \end{array}\right)}{\left(\begin{array}{c} N+N^{0}+N^{1}-1\\ N \end{array}\right)}.$$ (12)

This is the probability of finding the network with *k* nodes in state 1 at equilibrium, and it is independent of the initial state. The other eigenvectors, corresponding to *λ*_*r*_ ≠ 1, can also be calculated, and are also related to hypergeometric functions [18–19]. Although these eigenvectors provide a complete description of the dynamics of the network (see Eq 10), they are not particularly illuminating as we are interested in understanding the asymptotic behavior of the system (*λ*_0_ = 1).

In the thermodynamic limit *N* → ∞, we can define continuous variables *v* = *k*/*N*, *n*^0^ = *N*^0^/*N* and *n*^1^ = *N*^1^/*N* and approximate the asymptotic distribution presented in Eq 12 by a Gaussian $\rho \left(v\right)=1/\sqrt{2\pi \sigma^{2}}\rho_{0}e^{\left[-{\left(v-\mu \right)}^{2}/2\sigma^{2}\right]}$ with mean $\mu =n^{1}/\left(n^{0}+n^{1}\right)=N^{1}/\left(N^{0}+N^{1}\right)$ and variance

$$\sigma^{2}=\frac{n^{0}n^{1}\left(1+n^{0}+n^{1}\right)}{N{\left(n^{0}+n^{1}\right)}^{3}}=\mu \left(1-\mu \right)\left(\frac{1}{N^{0}+N^{1}}+\frac{1}{N}\right)$$ (13)

In the limit where *n*^0^, *n*^1^ ≫ 1, the width depends only on the ratio *α* = *n*^0^/*n*^1^ and is given by $\sqrt{\alpha /N}/\left(1+\alpha \right)$. In particular, for *n*^0^, *n*^1^ ≫ 1, the width tends to $1/\left(2\sqrt{N}\right)$.

While the model solved above was stated in terms of non-negative integer influence parameters *N*^0^, *N*^1^, it can be generalized to a model where the external influence parameters *N*^0^, *N*^1^ are real numbers. In this case, the solution in Eq 12 remains the same, with the difference that factorials must be replaced by gamma functions. Since the numbers *N*^0^/(*N* + *N*^0^ + *N*^1^ − 1) and *N*^1^/(*N* + *N*^0^ + *N*^1^ − 1) represent the probabilities that a free node (nonpartisan voter) copies one of the frozen nodes (partisan voters), small (large) values of *N*^0^ and *N*^1^ can be interpreted as representing a weak (strong) connection between the free nodes and the external system containing the frozen nodes. The external system can be thought of as a reservoir that affects the network but is not affected by it.

### Model behavior

**Fig 6:**
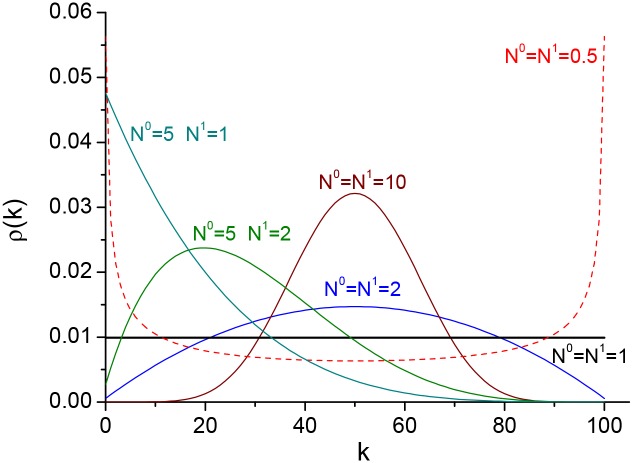
Stationary distributions for different values of *N*^0^ and *N*^1^. Probability distributions of finding the network with *k* nodes in state 1 at equilibrium for different values of *N*^0^ and *N*^1^. The number of variable nodes is *N* = 500.

Fig 6 shows examples of the distribution *ρ*(*k*) for a network with *N* = 500 and various values of *N*^0^ and *N*^1^. As we move around in the (*N*^0^, *N*^1^)-parameter space, we observe different types of behavior, which is characteristic of a first-order phase transition.

For *N*^0^ = *N*^1^ = 1, we obtain *ρ*(*k*) = 1/(*N* + 1) for all values of *N*, i.e. *N*^0^ = *N*^1^ = 1 is the *critical value* of this model. In this case, all states *S*_*k*_ are equally likely and the system executes a random walk through the state space. In the limit *N* → ∞, *N*^0^ = *N*^1^ = 1 marks the transition between disordered and ordered states.

For *N*^0^, *N*^1^ > 1, we obtain skewed unimodal distributions with peak at *N*^1^/(*N*^0^ + *N*^1^) corresponding to the fraction of voters in the network that voted for candidate 1. If *N*^1^ > *N*^0^, the majority of votes go to candidate 1, and if *N*^0^ > *N*^1^ the majority of votes go to candidate 0. We note that the estimation of the influence parameters *N*^0^, *N*^1^, based on almost a century of US presidential election data, predominantly falls within this regime. For *N*^0^, *N*^1^ ≫ 1, *ρ*(*k*) resembles a Gaussian distribution, and if *N*^0^ = *N*^1^ about half the voters vote for candidate 0 and half the voters vote for candidate 1, similarly to a magnetic material at high temperatures.

For *N*^0^, *N*^1^ < 1—the bistable (hysteresis) region—we obtain bimodal distributions in which either of the two network phases can exist, similar to the magnetization state in the Ising model below the critical temperature. For *N*^0^, *N*^1^ ≪ 1, the distribution peaks at all nodes 0 or all nodes 1, similar to a magnetized state at low temperatures.

Finally, for *N*^1^ > 1, *N*^0^ < 1 or *N*^1^ < 1, *N*^0^ > 1, we obtain unimodal distributions with peaks at all nodes 1 or all nodes 0, respectively.

### Other network topologies

Although the stationary vote-share distribution given by Eq 12 is obtained assuming fully connected networks, it was shown in [18–19] that our exact results are excellent approximations for other networks, including random, regular lattice, scale-free, and small world networks. These approximations can be useful, for example, if our model is applied to a network constructed based on online social networks or commuting networks. For these networks, which are not fully connected, the effect of the frozen nodes is amplified and can be quantified as follows: the probability that a free node copies a frozen node is *P*_*i*_ = (*N*^0^ + *N*^1^)/(*N*^0^ + *N*^1^ + *k*_*i*_) where *k*_*i*_ is the degree of the node. We can then define effective numbers of frozen nodes in the corresponding fully connected network, *N*^0*ef*^ and *N*^1*ef*^, as being the values for which

$$\frac{\left(N^{0ef}+N^{1ef}\right)}{\left(N^{0ef}+N^{1ef}+N-1\right)}=\sum_{k}\frac{\left(N^{0}+N^{1}\right)}{\left(N^{0}+N^{1}+k\right)}f\left(k\right)$$ (14)

$$\frac{N^{1ef}}{\left(N^{0ef}+N^{1ef}\right)}=\frac{N^{1}}{\left(N^{0}+N^{1}\right)}$$ (15)

where the term on the right-hand side in Eq 14 is the expectation with respect to the degree distribution *f*(*k*), and the term on the left-hand side is the probability that a free node copies a frozen node in the corresponding fully connected network. Eq 15 is the mean field boundary condition. For well-behaved distributions, *N*^0*ef*^ and *N*^1*ef*^ can be obtained in terms of central moments of the degree distribution by expanding the right-hand side in Eq 14 around the average degree 〈*k*〉 of the real network, as follows:

$$\frac{\left(N^{0ef}+N^{1ef}\right)}{\left(N^{0ef}+N^{1ef}+N-1\right)}=\sum_{{}_{n}}{\left(-1\right)}^{n}\frac{\left(N^{0}+N^{1}\right)}{{\left(N^{0}+N^{1}+k\right)}^{\left(n+1\right)}}\mu_{n}$$ (16)

where *μ*_*n*_ = ∑(k − 〈k〉)^*n*^ f(k) are the central moments of the distribution *f*(*k*). For example, using only the first term in the Taylor expansion gives (*N*^0*ef*^ + *N*^1*ef*^)/(*N*^0*ef*^ + *N*^1*ef*^+*N* − 1) = (*N*^0^ + *N*^1^)/(*N*^0^ + *N*^1^ + 〈*k*〉). This leads to

$$N^{0ef}=fN^{0}\;\;\;\;\;\;\;\;\;\;\;\;\;\;\;N^{1ef}=fN^{1}$$

where *f* = (*N* − 1)/〈*k*〉. Therefore, as the network acquires more internal connections and 〈*k*〉 increases, the effective values *N*^0*ef*^ and *N*^1*ef*^ decrease.

## Supporting information

S1 MovieHotspots of social contagion: 92 years of presidential elections.S1 Movie shows colored maps that reflect the significance (p-value) of local concentration of social influence for each state. The p-values for each state are derived from a random permutation test of local clustering using the Getis-Ord Local $G_{i}^{*}$ statistic. This analysis was performed with a contiguity spatial weight matrix that indicates whether states share a boundary or not. The variable of concern is the social influence index calculated using Eq 7 in the main text. Low p-values (p-value≤0.1) indicate statistically significant high levels of social influence at a state and its surrounding neighbors (hot spots). High p-values (p-value≥0.9) indicate statistically significant low levels of social influence at a state and its surrounding neighbors (cold spots).(MOV)

S2 Movie Social influence topography of the United States: 1920–2012. S2 Movie shows maps of social influence for all election years. The colored areas are derived from the social influence index calculated using Eq 7 in the main text.
(MOV)

S1 Fig Testing for a break in the level of social influence using the Mann-Whiney U-test. The Mann—Whitney U-test is a nonparametric test that assesses whether one of two random variables is stochastically larger than the other. Given a time-series of social influence from 1920 to 2012, we define for each election year, *y*, two samples of social influence: from 1920 to *y*−4, and from *y* to 2012. We apply the Mann—Whitney U-test for these two samples, and calculate the corresponding *p*-value. The optimal break date is the date that achieves the minimum *p*-value over all potential breaks within the range 1920–2012 (marked by a red circle in the above curve, plotted in a linear-log scale).
(TIF)

S2 FigHot spot analysis of social influence: 1920 US presidential election.The colored areas reflect the significance (p-value) of local concentration of social influence for each state. The p-values for each state are derived from a random permutation test of local clustering using the Getis-Ord Local $G_{i}^{*}$ statistic (see Fig 5 in main text for details).(TIF)

S3 FigHot spot analysis of social influence: 1924 US presidential election.The colored areas reflect the significance (p-value) of local concentration of social influence for each state. The p-values for each state are derived from a random permutation test of local clustering using the Getis-Ord Local $G_{i}^{*}$ statistic (see Fig 5 in main text for details).(TIF)

S4 FigHot spot analysis of social influence: 1928 US presidential election.The colored areas reflect the significance (p-value) of local concentration of social influence for each state. The p-values for each state are derived from a random permutation test of local clustering using the Getis-Ord Local $G_{i}^{*}$ statistic (see Fig 5 in main text for details).(TIF)

S5 FigHot spot analysis of social influence: 1932 US presidential election.The colored areas reflect the significance (p-value) of local concentration of social influence for each state. The p-values for each state are derived from a random permutation test of local clustering using the Getis-Ord Local $G_{i}^{*}$ statistic (see Fig 5 in main text for details).(TIF)

S6 FigHot spot analysis of social influence: 1936 US presidential election.The colored areas reflect the significance (p-value) of local concentration of social influence for each state. The p-values for each state are derived from a random permutation test of local clustering using the Getis-Ord Local $G_{i}^{*}$ statistic (see Fig 5 in main text for details).(TIF)

S7 FigHot spot analysis of social influence: 1940 US presidential election.The colored areas reflect the significance (p-value) of local concentration of social influence for each state. The p-values for each state are derived from a random permutation test of local clustering using the Getis-Ord Local $G_{i}^{*}$ statistic (see Fig 5 in main text for details).(TIF)

S8 FigHot spot analysis of social influence: 1944 US presidential election.The colored areas reflect the significance (p-value) of local concentration of social influence for each state. The p-values for each state are derived from a random permutation test of local clustering using the Getis-Ord Local $G_{i}^{*}$ statistic (see Fig 5 in main text for details).(TIF)

S9 FigHot spot analysis of social influence: 1948 US presidential election.The colored areas reflect the significance (p-value) of local concentration of social influence for each state. The p-values for each state are derived from a random permutation test of local clustering using the Getis-Ord Local $G_{i}^{*}$ statistic (see Fig 5 in main text for details).(TIF)

S10 FigHot spot analysis of social influence: 1952 US presidential election.The colored areas reflect the significance (p-value) of local concentration of social influence for each state. The p-values for each state are derived from a random permutation test of local clustering using the Getis-Ord Local $G_{i}^{*}$ statistic (see Fig 5 in main text for details).(TIF)

S11 FigHot spot analysis of social influence: 1956 US presidential election.The colored areas reflect the significance (p-value) of local concentration of social influence for each state. The p-values for each state are derived from a random permutation test of local clustering using the Getis-Ord Local $G_{i}^{*}$ statistic (see Fig 5 in main text for details).(TIF)

S12 FigHot spot analysis of social influence: 1960 US presidential election.The colored areas reflect the significance (p-value) of local concentration of social influence for each state. The p-values for each state are derived from a random permutation test of local clustering using the Getis-Ord Local $G_{i}^{*}$ statistic (see Fig 5 in main text for details).(TIF)

S13 FigHot spot analysis of social influence: 1964 US presidential election.The colored areas reflect the significance (p-value) of local concentration of social influence for each state. The p-values for each state are derived from a random permutation test of local clustering using the Getis-Ord Local $G_{i}^{*}$ statistic (see Fig 5 in main text for details).(TIF)

S14 FigHot spot analysis of social influence: 1968 US presidential election.The colored areas reflect the significance (p-value) of local concentration of social influence for each state. The p-values for each state are derived from a random permutation test of local clustering using the Getis-Ord Local $G_{i}^{*}$ statistic (see Fig 5 in main text for details).(TIF)

S15 FigHot spot analysis of social influence: 1972 US presidential election.The colored areas reflect the significance (p-value) of local concentration of social influence for each state. The p-values for each state are derived from a random permutation test of local clustering using the Getis-Ord Local $G_{i}^{*}$ statistic (see Fig 5 in main text for details).(TIF)

S16 FigHot spot analysis of social influence: 1976 US presidential election.The colored areas reflect the significance (p-value) of local concentration of social influence for each state. The p-values for each state are derived from a random permutation test of local clustering using the Getis-Ord Local $G_{i}^{*}$ statistic (see Fig 5 in main text for details).(TIF)

S17 FigHot spot analysis of social influence: 1980 US presidential election.The colored areas reflect the significance (p-value) of local concentration of social influence for each state. The p-values for each state are derived from a random permutation test of local clustering using the Getis-Ord Local $G_{i}^{*}$ statistic (see Fig 5 in main text for details).(TIF)

S18 FigHot spot analysis of social influence: 1984 US presidential election.The colored areas reflect the significance (p-value) of local concentration of social influence for each state. The p-values for each state are derived from a random permutation test of local clustering using the Getis-Ord Local $G_{i}^{*}$ statistic (see Fig 5 in main text for details).(TIF)

S19 FigHot spot analysis of social influence: 1988 US presidential election.The colored areas reflect the significance (p-value) of local concentration of social influence for each state. The p-values for each state are derived from a random permutation test of local clustering using the Getis-Ord Local $G_{i}^{*}$ statistic (see Fig 5 in main text for details).(TIF)

S20 FigHot spot analysis of social influence: 1992 US presidential election.The colored areas reflect the significance (p-value) of local concentration of social influence for each state. The p-values for each state are derived from a random permutation test of local clustering using the Getis-Ord Local $G_{i}^{*}$ statistic (see Fig 5 in main text for details).(TIF)

S21 FigHot spot analysis of social influence: 1996 US presidential election.The colored areas reflect the significance (p-value) of local concentration of social influence for each state. The p-values for each state are derived from a random permutation test of local clustering using the Getis-Ord Local $G_{i}^{*}$ statistic (see Fig 5 in main text for details).(TIF)

S22 FigHot spot analysis of social influence: 2000 US presidential election.The colored areas reflect the significance (p-value) of local concentration of social influence for each state. The p-values for each state are derived from a random permutation test of local clustering using the Getis-Ord Local $G_{i}^{*}$ statistic (see Fig 5 in main text for details).(TIF)

S23 FigHot spot analysis of social influence: 2004 US presidential election.The colored areas reflect the significance (p-value) of local concentration of social influence for each state. The p-values for each state are derived from a random permutation test of local clustering using the Getis-Ord Local $G_{i}^{*}$ statistic (see Fig 5 in main text for details).(TIF)

S24 FigHot spot analysis of social influence: 2008 US presidential election.The colored areas reflect the significance (p-value) of local concentration of social influence for each state. The p-values for each state are derived from a random permutation test of local clustering using the Getis-Ord Local $G_{i}^{*}$ statistic (see Fig 5 in main text for details).(TIF)

S25 FigHot spot analysis of social influence: 2012 US presidential election.The colored areas reflect the significance (p-value) of local concentration of social influence for each state. The p-values for each state are derived from a random permutation test of local clustering using the Getis-Ord Local *$G_{i}^{*}$* statistic (see Fig 5 in main text for details).(TIF)

S1 Table Results of random permutation tests of spatial autocorrelation using Moran’s *I* statistic. This analysis was performed with a contiguity spatial weight matrix (row normalized) that indicates whether states share a boundary or not. The variable of concern is the social influence index calculated using Eq 7 in the main text. The observed Moran’s *I* statistics are shown in the second column and the corresponding significance levels (p-values) of the tests are shown in the third column. The random permutation tests suggest the presence of significant positive spatial autocorrelation as indicated by the level of significance (p-value) shown in the third column.
(TIF)

S2 Table Results of random permutation tests of spatial clustering using Getis-Ord General G* statistic. This analysis was performed with a contiguity spatial weight matrix that indicates whether states share a boundary or not. The variable of concern is the social influence index calculated using Eq 7 in the main text. The observed Getis-Ord General G* statistics and significance levels (p-values) of the tests are shown in the second and third columns, respectively. The tests indicate that social influence is significantly concentrated in space as shown by the significance levels (p-value) in the third column. For all election years, the observed Getis-Ord General G* is larger than the expected General G*, indicating that the spatial distribution of high social influence values is more spatially clustered than would be expected if underlying spatial processes were truly random.
(TIF)

## References

[1] Galam S (2005) Local dynamics vs. social mechanisms: A unifying frame. *Europhysics Letters* 70(6): 705–711.

[2] San Miguel M, Eguiluz VM, Toral R, Klemm K (2005) Binary and multivariate stochastic models of consensus formation. *Computing in Science & Engineering*, 7(6): 67–73.

[3] Castellano C, Fortunato S, and Loreto V (2009) Statistical physics of social dynamics. *Rev Mod Phys* 81(2): 591–646.

[4] Costa LDF, Oliveira ON, Travieso G, Rodrigues FA, Villas Boas PR, Antiqueira L, et al (2011) Analyzing and modeling real-world phenomena with complex networks: a survey of applications. *Adv Phys* 60(3): 329–412.

[5] Galam S (2012) *Sociophysics*: *a physicist's modeling of psycho-political phenomena* (Springer, New York).

[6] Helbing D, Brockmann D, Chadefaux T, Donnay K, Blanke U, Woolley-Meza O, et al (2015) Saving human lives: what complexity science and information systems can contribute. *J Stat Phys* 158(3): 735–781. 10.1007/s10955-014-1024-9 26074625 PMC4457089

[7] Gonçalves B, Perra N (2015) *Social Phenomena*: *From Data to Models* (Springer, New York).

[8] Aral S, Walker D (2012) Identifying influential and susceptible members of social networks. *Science* 337(6092): 337–341. 10.1126/science.1215842 22722253

[9] Centola D (2010) The spread of behavior in an online social network experiment. *Science* 329(5996): 1194–1197. 10.1126/science.1185231 20813952

[10] Fowler JH, Christakis NA (2010) Cooperative behavior cascades in human social networks. *Proc Natl Acad Sci USA* 107(12): 5334–5338. 10.1073/pnas.0913149107 20212120 PMC2851803

[11] Bond RM, Fariss CJ, Jones JJ, Kramer AD, Marlow C, Settle JE, et al (2012) A 61-million-person experiment in social influence and political mobilization. *Nature* 489(7415): 295–298. 10.1038/nature11421 22972300 PMC3834737

[12] Nickerson DW (2008) Is voting contagious? evidence from two field experiments. *Am Polit Sci Rev* 102(01): 49–57.

[13] Alexander C, Piazza M, Mekos D, Valente T (2001) Peers, schools, and adolescent cigarette smoking. *J Adolesc Health* 29(1): 22–30. 11429302 10.1016/s1054-139x(01)00210-5

[14] Valente TW (2005) Network models and methods for studying the diffusion of innovations *Models and Methods in Social Network Analysis*, eds Carrington PJ, Scott J, Wasserman S (Cambridge University Press, New York), pp 98–116.

[15] Christakis NA, Fowler JH (2007) The spread of obesity in a large social network over 32 years. *N Engl J Med* 357(4): 370–379. 10.1056/NEJMsa066082 17652652

[16] Fowler JH, Christakis NA (2008) Dynamic spread of happiness in a large social network: longitudinal analysis over 20 years in the Framingham Heart Study. *Bmj* 337: a2338 10.1136/bmj.a2338 19056788 PMC2600606

[17] Liggett T (2012) *Interacting Particle Systems* (Springer, New York).

[18] Chinellato DD, de Aguiar MAM, Epstein IR, Braha D, Bar Yam Y (2007) Dynamical response of networks under external perturbations: exact results. Preprint. Available: arXiv:0705.4607v2. Accessed 26 August 2016.

[19] Chinellato DD, Epstein IR, Braha D, Bar Yam Y, de Aguiar MAM (2015) Dynamical response of networks under external perturbations: exact results. *J Stat Phys* 159(2): 221–230.

[20] Campbell A (1980) *The American Voter* (University of Chicago Press, Chicago).

[21] Lazarsfeld PF, Gaudet H, Berelson B (1965) *The People's Choice*: *How the Voter Makes Up His Mind in a Presidential Campaign* (Columbia University Press, New York).

[22] Berelson BR, Lazarsfeld PF, McPhee WN (1954) *Voting*: *A Study of Opinion Formation in a Presidential Election* (University of Chicago Press, Chicago).

[23] Downs A (1957) *An Economic Theory of Democracy* (Harper, New York).

[24] Beck PA, Dalton RJ, Greene S, Huckfeldt R (2002) The social calculus of voting: Interpersonal, media, and organizational influences on presidential choices. *Am Polit Sci Rev* 96 (1): 57–73.

[25] Kenny CB (1992) Political participation and effects from the social environment. *Am J Polit Sci* 36(1): 259–267.

[26] Huckfeldt RR, Sprague J. (1995) *Citizens*, *Politics and Social Communication*: *Information and Influence in an Election Campaign* (Cambridge University Press, New York).

[27] McClurg SD (2004) Indirect mobilization the social consequences of party contacts in an election campaign. *Am Polit Res* 32(4): 406–443.

[28] Huckfeldt R, Sprague J (1991) Discussant effects on vote choice: intimacy, structure, and interdependence. *J Polit* 53(01): 122–158.

[29] Mobilia M (2003) Does a single zealot affect an infinite group of voters? *Phys Rev Lett*, 91(2): 028701 10.1103/PhysRevLett.91.028701 12906515

[30] Galam S, Jacobs F (2007) The role of inflexible minorities in the breaking of democratic opinion dynamics. *Physica A* 381: 366–376.

[31] Harmon D, Lagi M, de Aguiar MA, Chinellato DD, Braha D, Epstein IR, et al (2015) Anticipating economic market crises using measures of collective panic. *PloS one* 10(7): e0131871 10.1371/journal.pone.0131871 26185988 PMC4506134

[32] Acemoglu D, Como G, Fagnani F, Ozdaglar A (2013) Opinion fluctuations and disagreement in social networks. *Mathematics of Operations Research* 38(1): 1–27.

[33] Yildiz E, Acemoglu D, Ozdaglar AE, Saberi A, Scaglione A (2013) Discrete opinion dynamics with stubborn agents. *ACM Transactions on Economics and Computation* 1(4): 1–30.

[34] Wu Y, Shen J (2012) Opinion dynamics with stubborn vertices. *Electronic Journal of Linear Algebra* 23(1): 790–800.

[35] Galam S (2016) Stubbornness as an unfortunate key to win a public debate: an illustration from sociophysics. *Mind &* *Society* 15(1): 117–130.

[36] Xie J, Sreenivasan S, Korniss G, Zhang W, Lim C, Szymanski BK (2011) Social consensus through the influence of committed minorities. *Phys Rev E* 84(1): 011130. 10.1103/PhysRevE.84.011130 21867136

[37] Xie J, Emenheiser J, Kirby M, Sreenivasan S, Szymanski BK, Korniss G (2012) Evolution of opinions on social networks in the presence of competing committed groups. *PLoS ONE* 7(3): e33215 10.1371/journal.pone.0033215 22448238 PMC3308977

[38] Singh P, Sreenivasan S, Szymanski BK, Korniss G (2012) Accelerating consensus on coevolving networks: The effect of committed individuals. *Phys Rev E* 85: 046104. 10.1103/PhysRevE.85.046104 22680535

[39] Kinder DR, Iyengar S (1987) *News that Matters* (University of Chicago Press, Chicago).

[40] Just MR (1996) *Crosstalk*: *Citizens*, *Candidates*, *and the Media in a Presidential Campaign* (University of Chicago Press, Chicago).

[41] Dalton RJ, Beck PA, Huckfeldt R (1998) Partisan cues and the media: Information flows in the 1992 presidential election. *Am Polit Sci Rev* 92(01): 111–126.

[42] Gilens M, Vavreck L, Cohen M (2007) The mass media and the public's assessments of presidential candidates, 1952–2000. *J Polit* 69(4): 1160–1175.

[43] Armoudian M, Crigler AN (2010) Constructing the vote: Media effects in a constructionist model *The Oxford Handbook of American Elections and Political Behavior*, eds Leighley JE (Oxford University Press, Oxford), pp 300–325.

[44] Hillygus S (2010) Campaign effects on vote choice *The Oxford Handbook of American Elections and Political Behavior* eds Leighley JE (Oxford University Press, Oxford), pp 326–345.

[45] Sah RK (1991) Social osmosis and patterns of crime: A dynamic economic analysis. *J Polit Econ* 99(6): 1272–1295.

[46] Jones AM (1994) Health, addiction, social interaction and the decision to quit smoking. *J Health Econ* 13(1): 93–110. 10134441 10.1016/0167-6296(94)90006-x

[47] Braha D (2012) Global civil unrest: contagion, self-organization, and prediction. *PloS one* 7(10): e48596 10.1371/journal.pone.0048596 23119067 PMC3485346

[48] Costa Filho RN, Almeida MP, Andrade JS, Moreira JE (1999) Scaling behavior in a proportional voting process. *Phys Rev E* 60(1): 1067. 10.1103/physreve.60.1067 11969855

[49] Bernardes AT, Stauffer D, Kertesz J (2002) Election results and the Sznajd model on Barabasi network. *Eur*. *Phys*. *J*. *B* 25(1): 123–127.

[50] Costa Filho RN, Almeida MP, Moreira JE, Andrade JS (2003) Brazilian elections: voting for a scaling democracy. *Physica A* 322: 698–700.

[51] Lyra ML, Costa UMS, Costa Filho RN, Andrade JS Jr (2003) Generalized Zipf's law in proportional voting processes. *Europhys*. *Lett*. 62(1): 131–137.

[52] Fortunato S, Castellano C (2007) Scaling and universality in proportional elections. *Phys Rev Lett* 99(13): 138701 10.1103/PhysRevLett.99.138701 17930647

[53] Araújo NA, Andrade JS Jr, Herrmann HJ (2010) Tactical voting in plurality elections. *PLoS One* 5(9): e12446 10.1371/journal.pone.0012446 20856800 PMC2939874

[54] Borghesi C, Bouchaud J-P (2010) Spatial correlations in vote statistics: a diffusive field model for decision-making. *Eur Phys J B* 75(3): 395–404.

[55] Borghesi C, Raynal J-C, Bouchaud J-P (2012) Election turnout statistics in many countries: similarities, differences, and a diffusive field model for decision-making. *PloS One* 7(5): e36289 10.1371/journal.pone.0036289 22615762 PMC3354000

[56] Chatterjee A, Mitrovic M, and Fortunato S (2013) Universality in voting behavior: an empirical analysis. *Sci*. *Rep*. 3: 1049 10.1038/srep01049 23308342 PMC3541511

[57] Mantovani MC, Ribeiro HV, Lenzi EK, Picoli S Jr, Mendes RS (2013) Engagement in the electoral processes: scaling laws and the role of political positions. *Phys Rev E* 88(2): 024802. 10.1103/PhysRevE.88.024802 24032971

[58] Fernández-Gracia J, Suchecki K, Ramasco JJ, San Miguel M, Eguíluz VM (2014) Is the Voter Model a model for voters? *Phys Rev Lett* 112(15): 158701 10.1103/PhysRevLett.112.158701 24785078

[59] Palombi F, Toti S (2014) Stochastic dynamics of the multi-state voter model over a network based on interacting cliques and zealot candidates. *J Stat Phys* 156(2): 336–367.

[60] Latané B (1981) The psychology of social impact. *American psychologist* 36(4): 343–356.

[61] Nowak A, Szamrej J, Latané B (1990) From private attitude to public opinion: A dynamic theory of social impact. *Psychological Review* 97(3): 362–376.

[62] Lyst J, Kacperski K, Schweitzer F (2002) Social impact models of opinion dynamics. *Annual reviews of computational physics* 9: 253–273.

[63] Boccara N (2007) Models of opinion formation: influence of opinion leaders. Preprint. Available: arXiv:0704.1790. Accessed 2 April 2017.

[64] Galam S (1997) Rational group decision making: A random field Ising model at T = 0. *Physica A*, 238(1–4): 66–80.

[65] Carletti T, Fanelli D, Grolli S, Guarino A (2006) How to make an efficient propaganda. *Europhysics Letters* 74(2): 222.

[66] Kuperman M, Zanette D (2002) Stochastic resonance in a model of opinion formation on small-world networks. *The European Physical Journal B* 26(3): 387–391.

[67] Tessone CJ, Toral R (2005) System size stochastic resonance in a model for opinion formation. *Physica A* 351(1): 106–116.

[68] González-Avella JC, Cosenza MG, Tucci K (2005) Nonequilibrium transition induced by mass media in a model for social influence. *Phys Rev E* 72(6): 065102. 10.1103/PhysRevE.72.065102 16485996

[69] Shibanai Y, Yasuno S, Ishiguro I (2001) Effects of global information feedback on diversity extensions to Axelrod's adaptive culture model. *Journal of Conflict Resolution* 45(1): 80–96.

[70] Mazzitello KI, Candia J, Dossetti V (2007) Effects of mass media and cultural drift in a model for social influence. *International Journal of Modern Physics C* 18(9): 1475–1482.

[71] Galam S (2005) Heterogeneous beliefs, segregation, and extremism in the making of public opinions. Phys Rev E 71(4): 046123. 10.1103/PhysRevE.71.046123 15903742

[72] Galam S (2016). The Trump phenomenon, an explanation from sociophysics. arXiv preprint arXiv:1609.03933.

[73] Galam S (2004) Contrarian deterministic effects on opinion dynamics: the hung elections scenario. *Physica A* 333: 453–460.

[74] Shao J, Havlin S, Stanley HE (2009) Dynamic opinion model and invasion percolation. *Phys Rev Lett* 103(1): 018701 10.1103/PhysRevLett.103.018701 19659181

[75] Li Q, Braunstein LA, Wang H, Shao J, Stanley HE, Havlin S (2013) Non-consensus opinion models on complex networks. *J Stat Phys* 151(1–2): 92–112.

[76] Mobilia M, Petersen A, Redner S (2007) On the role of zealotry in the voter model. *Journal of Statistical Mechanics*: *Theory and Experiment*, 2007(08): P08029.

[77] Galam S. (2002). Minority opinion spreading in random geometry. The European Physical Journal B-Condensed Matter and Complex Systems, 25(4), 403–406.

[78] Klimek P, Yegorov Y, Hanel R, Thurner S (2012) Statistical detection of systematic election irregularities. *Proc Natl Acad Sci USA* 109(41): 16469–16473. 10.1073/pnas.1210722109 23010929 PMC3478593

[79] Harmon D, de Aguiar MAM, Chinellato DD, Braha D, Epstein IR, Bar-Yam Y (2011) Predicting economic market crises using measures of collective panic. Preprint. Available: arXiv:1102.2620. Accessed 26 August 2016.

[80] de Aguiar MAM, Bar-Yam Y (2011) Moran model as a dynamical process on networks and its implications for neutral speciation. *Phys Rev E* 84(3): 031901. 10.1103/PhysRevE.84.031901 22060397

[81] Everitt BS and Hand DJ (1981) *Finite Mixture Distributions* (Chapman and Hall, New York).

[82] Felgueiras M, Santos R, and Martins JP (2014) Some results on Gaussian mixtures. *AIP Conf Proc* 1618: 523–526.

[83] For this research, we relied on county-level vote totals from CQ Press’ Voting and Elections Collection for every presidential election from 1920 through 2012.

[84] Kleiber C, Kotz S (2003) Statistical Size Distributions in Economics and Actuarial Sciences (John Wiley & Sons, New Jersey).

[85] Bryan FM (2010) New England *The Princeton Encyclopedia of American Political History* ed Kazin M (Princeton University Press, Princeton), pp 532–539.

[86] Lichtman AJ (2010) Elections and electoral eras *The Princeton Encyclopedia of American Political History* ed Kazin M (Princeton University Press, Princeton), pp 281–289.

[87] Bianco WT, Canon DT (2014) *American Politics Today* (WW Norton and Company, New York).

[88] Schaffner BF (2011) *Politics*, *Parties*, *and Elections in America* (Cengage Learning, Boston).

[89] Burnham WD (1970) *Critical Elections and the Mainspring of American Politics* (WW Norton, New York).

[90] Sundquist JL (2011) *Dynamics of the Party System*: *Alignment and Realignment of Political Parties in the United States*. Brookings Institution Press, Washington, DC).

[91] Wattenberg M (1994) The Decline of American Political Parties, 1952–1992. (Harvard University Press, Cambridge, MA).

[92] Putnam RD. (2001) Bowling Alone: The Collapse and Revival of American Community (Simon and Schuster, New York).

[93] Rubin RL (1981) Press, Party, and Presidency (WW Norton and Company, New York).

[94] Moran PA (1950) Notes on continuous stochastic phenomena. *Biometrika* 37(1/2): 17–23. 15420245

[95] Moran PA (1950) A test for the serial independence of residuals. *Biometrika* 37(1/2): 178–181. 15420264

[96] Getis A, Ord JK (1992) The analysis of spatial association by use of distance statistics. *Geographical analysis* 24(3): 189–206.

[97] Ord JK, Getis A (1995) Local spatial autocorrelation statistics: distributional issues and an application. *Geographical analysis* 27(4): 286–306.

[98] Krug SE, Raymond WK (1973) Personality differences across regions of the United States. *J Soc Psychol* 91(1): 73–79. 10.1080/00224545.1973.9922648 4749508

[99] Plaut VC, Markus HR, Lachman ME (2002) Place matters: consensual features and regional variation in American well-being and self. *J Pers Soc Psychol* 83(1): 160–184. 12088124

[100] Rentfrow PJ, Gosling SD, Jokela M, Stillwell DJ, Kosinski M, Potter J (2013) Divided we stand: Three psychological regions of the United States and their political, economic, social, and health correlates. *J Pers Soc Psychol* 105(6): 996–1012. 10.1037/a0034434 24128185

[101] Heppen J (2003) Racial and social diversity and US presidential election regions. *Prof Geogr* 55(2): 191–205.

[102] Hero RE (2000) Faces of Inequality: Social Diversity in American Politics (Oxford University Press, Oxford).

[103] Florida R (2002) The Rise of the Creative Class: and How It's Transforming Work, Leisure, Community and Everyday Life (Basic Books, New York).

[104] Scheffer M, Bascompte J, Brock WA, Brovkin V, Carpenter SR, Dakos V, et al (2009) Early-warning signals for critical transitions. *Nature* 461(7260): 53–59. 10.1038/nature08227 19727193

[105] Schelling TC (1971) Dynamic models of segregation. *J Math Sociol* 1(2): 143–186.

