# Supplementary figures and images for: Voting contagion: Modeling and analysis of a century of U.S. presidential elections
Source: PLoS One. 2017 May 18;12(5):e0177970. doi: 10.1371/journal.pone.0177970 (PMC5436881; doi:10.1371/journal.pone.0177970)

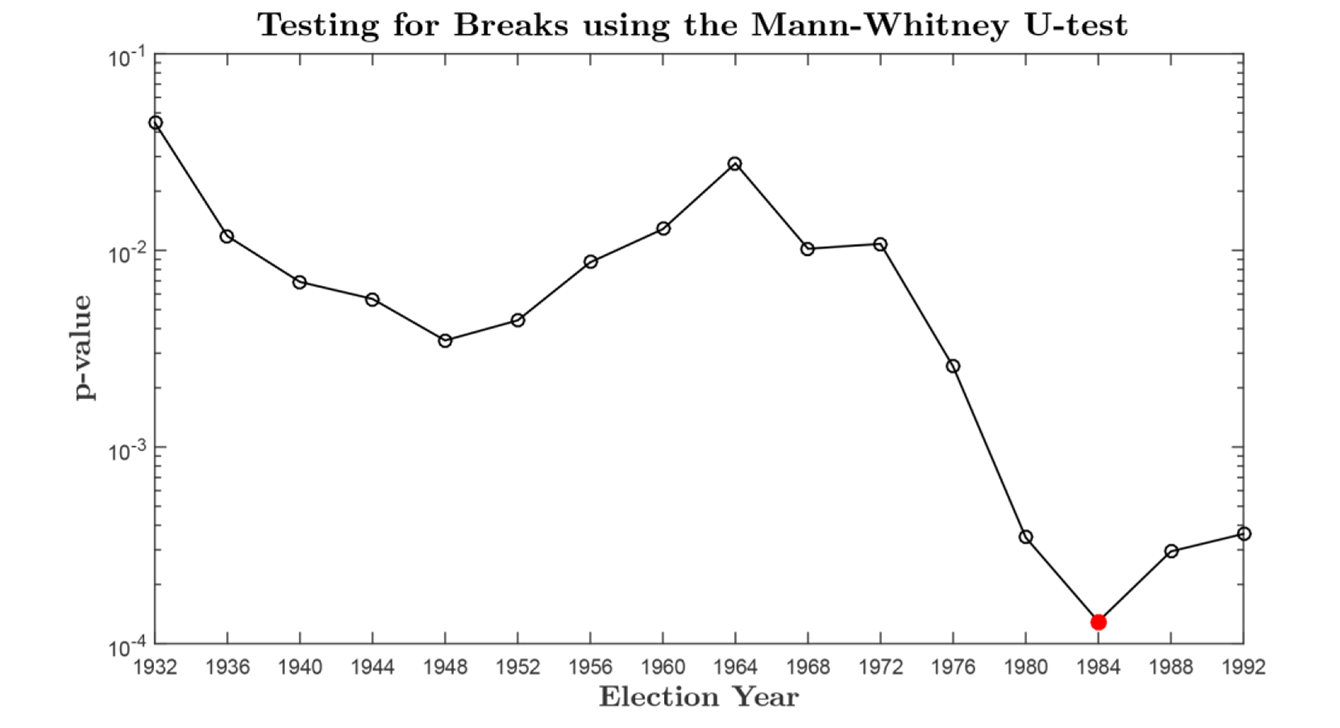

Supplement: S1 Fig — The Mann—Whitney U-test is a nonparametric test that assesses whether one of two random variables is stochastically larger than the other. Given a time-series of social influence from 1920 to 2012, we define for each election year, y, two samples of social influence: from 1920 to y−4, and from y to 2012. We apply the Mann—Whitney U-test for these two samples, and calculate the corresponding p-value. The optimal break date is the date that achieves the minimum p-value over all potential breaks within the range 1920–2012 (marked by a red circle in the above curve, plotted in a linear-log scale). (TIF) [file pone.0177970.s003.tif]

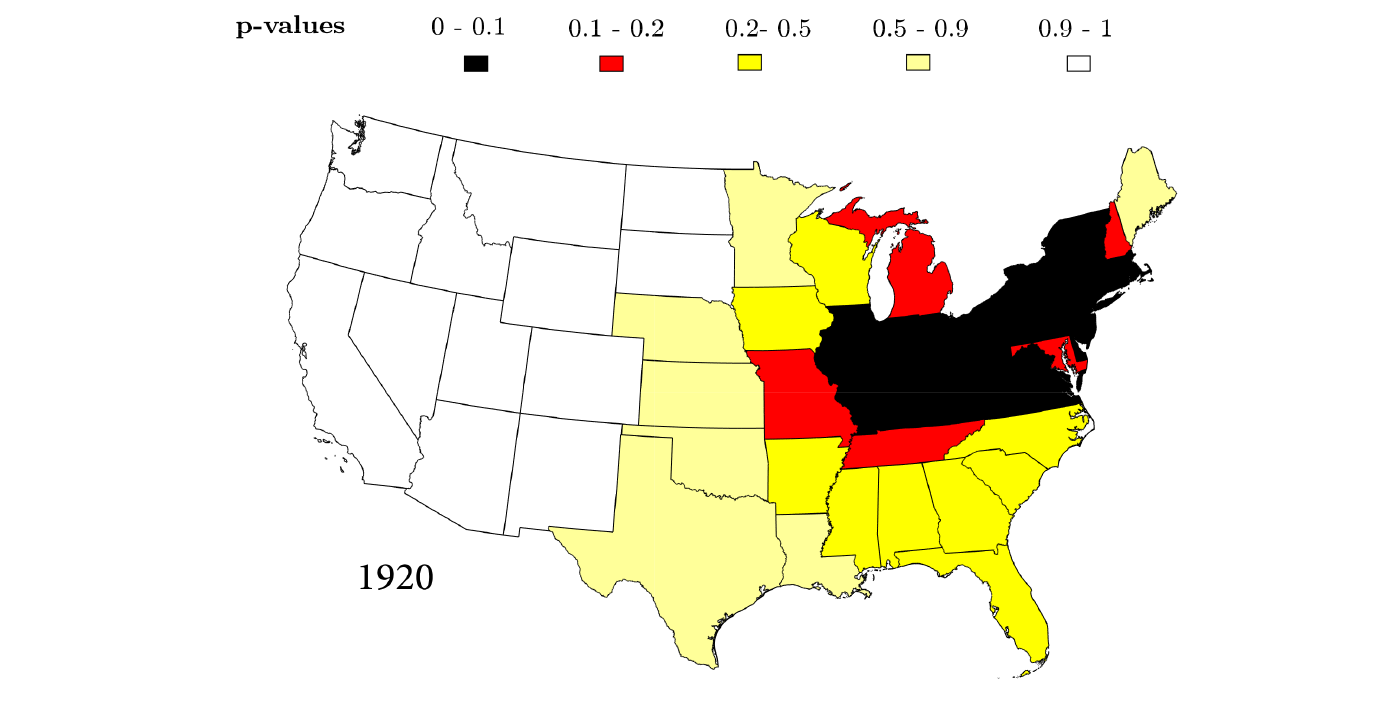

Supplement: S2 Fig — The colored areas reflect the significance (p-value) of local concentration of social influence for each state. The p-values for each state are derived from a random permutation test of local clustering using the Getis-Ord Local Gi* statistic (see Fig 5 in main text for details). (TIF) [file pone.0177970.s004.tif]

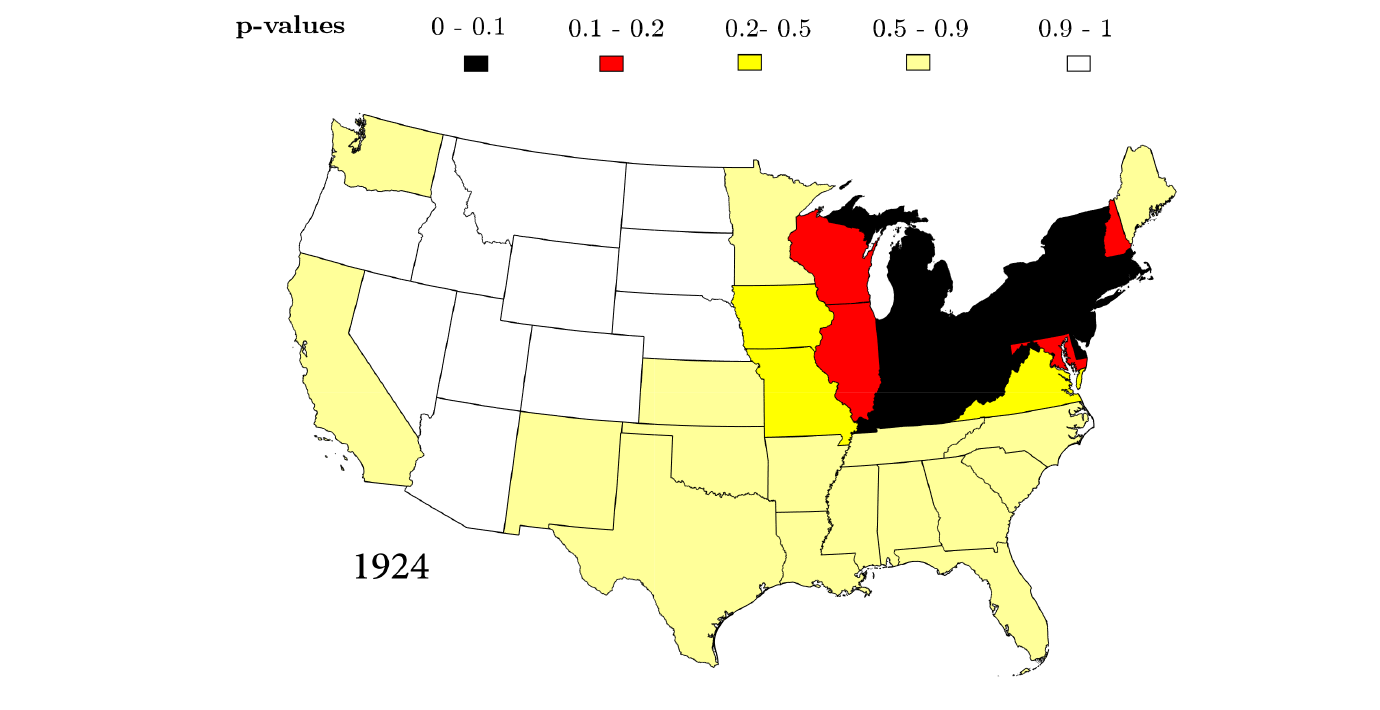

Supplement: S3 Fig — The colored areas reflect the significance (p-value) of local concentration of social influence for each state. The p-values for each state are derived from a random permutation test of local clustering using the Getis-Ord Local Gi* statistic (see Fig 5 in main text for details). (TIF) [file pone.0177970.s005.tif]

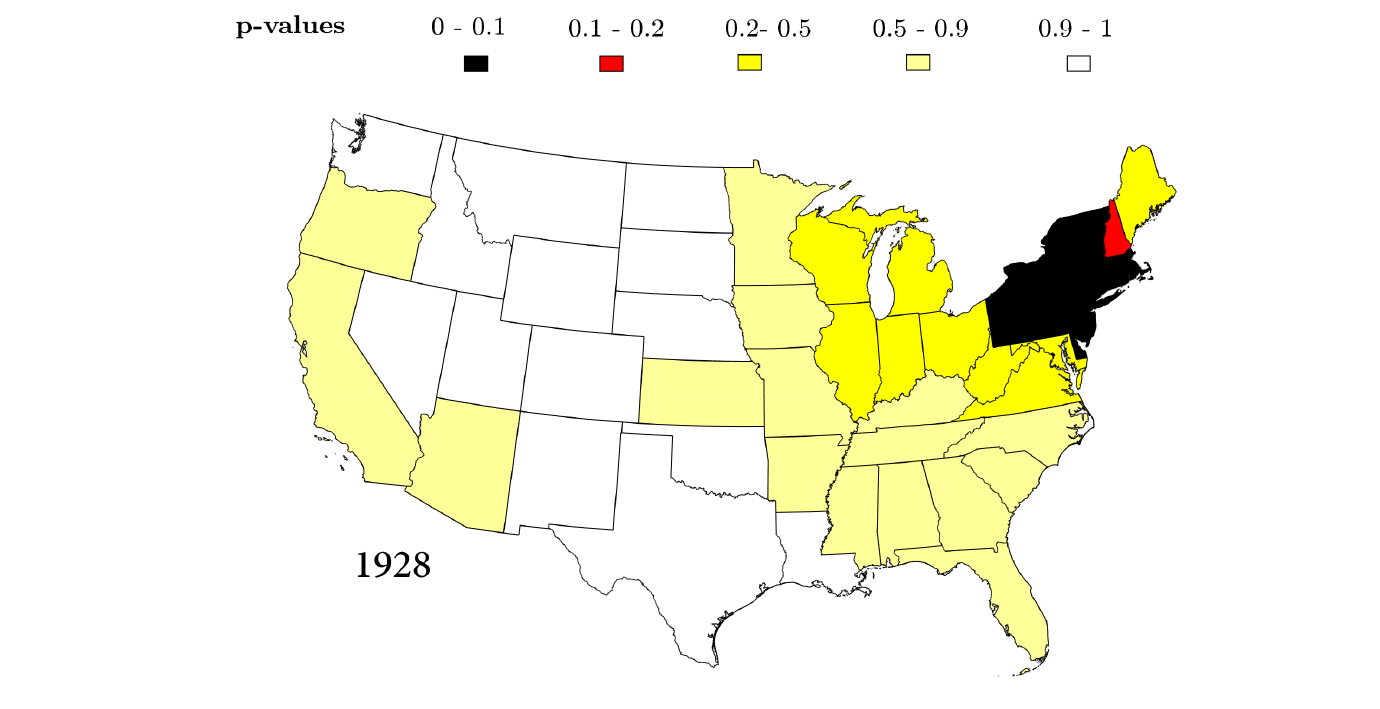

Supplement: S4 Fig — The colored areas reflect the significance (p-value) of local concentration of social influence for each state. The p-values for each state are derived from a random permutation test of local clustering using the Getis-Ord Local Gi* statistic (see Fig 5 in main text for details). (TIF) [file pone.0177970.s006.tif]

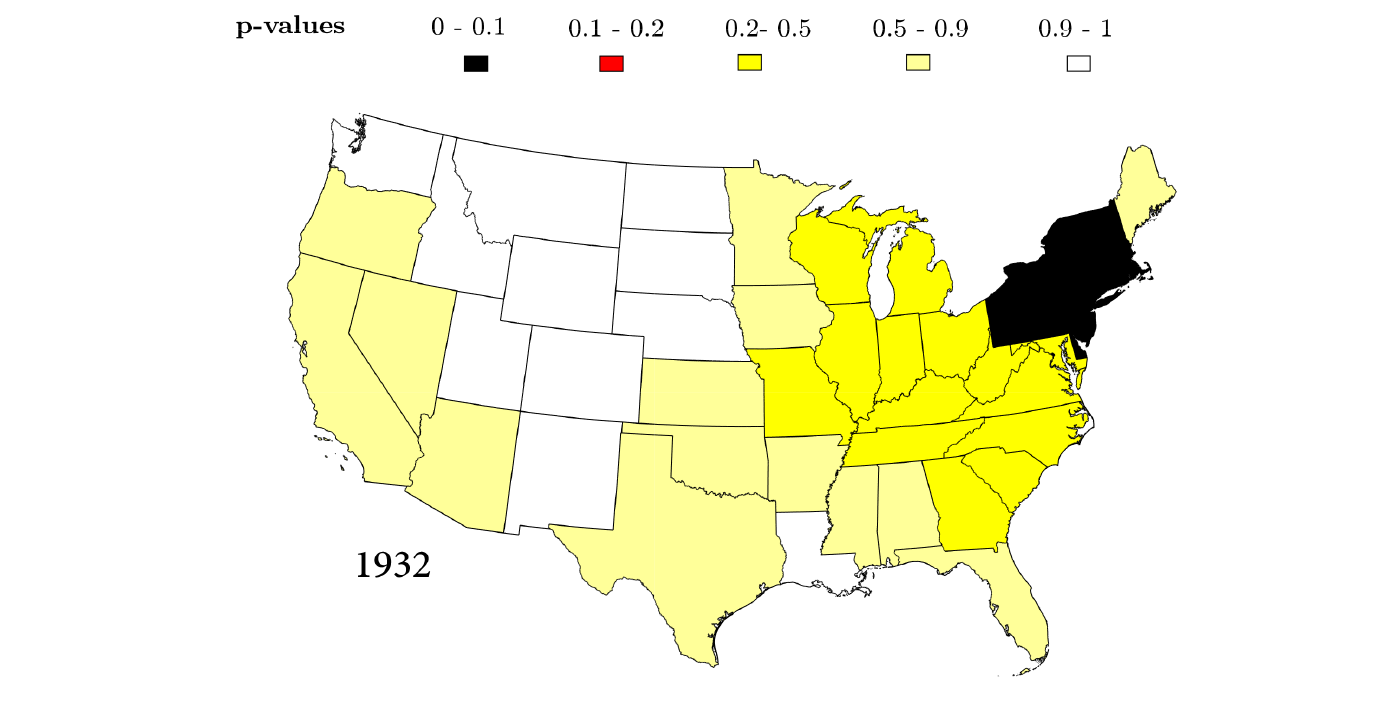

Supplement: S5 Fig — The colored areas reflect the significance (p-value) of local concentration of social influence for each state. The p-values for each state are derived from a random permutation test of local clustering using the Getis-Ord Local Gi* statistic (see Fig 5 in main text for details). (TIF) [file pone.0177970.s007.tif]

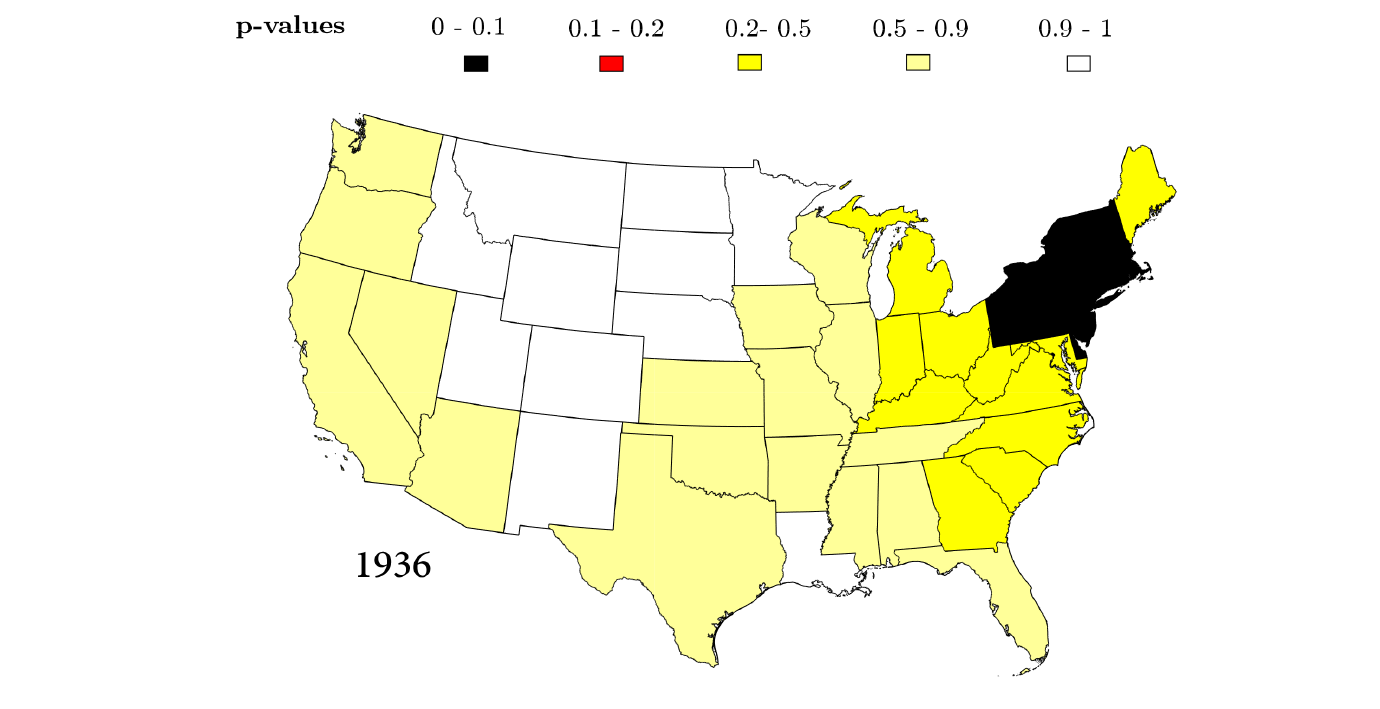

Supplement: S6 Fig — The colored areas reflect the significance (p-value) of local concentration of social influence for each state. The p-values for each state are derived from a random permutation test of local clustering using the Getis-Ord Local Gi* statistic (see Fig 5 in main text for details). (TIF) [file pone.0177970.s008.tif]

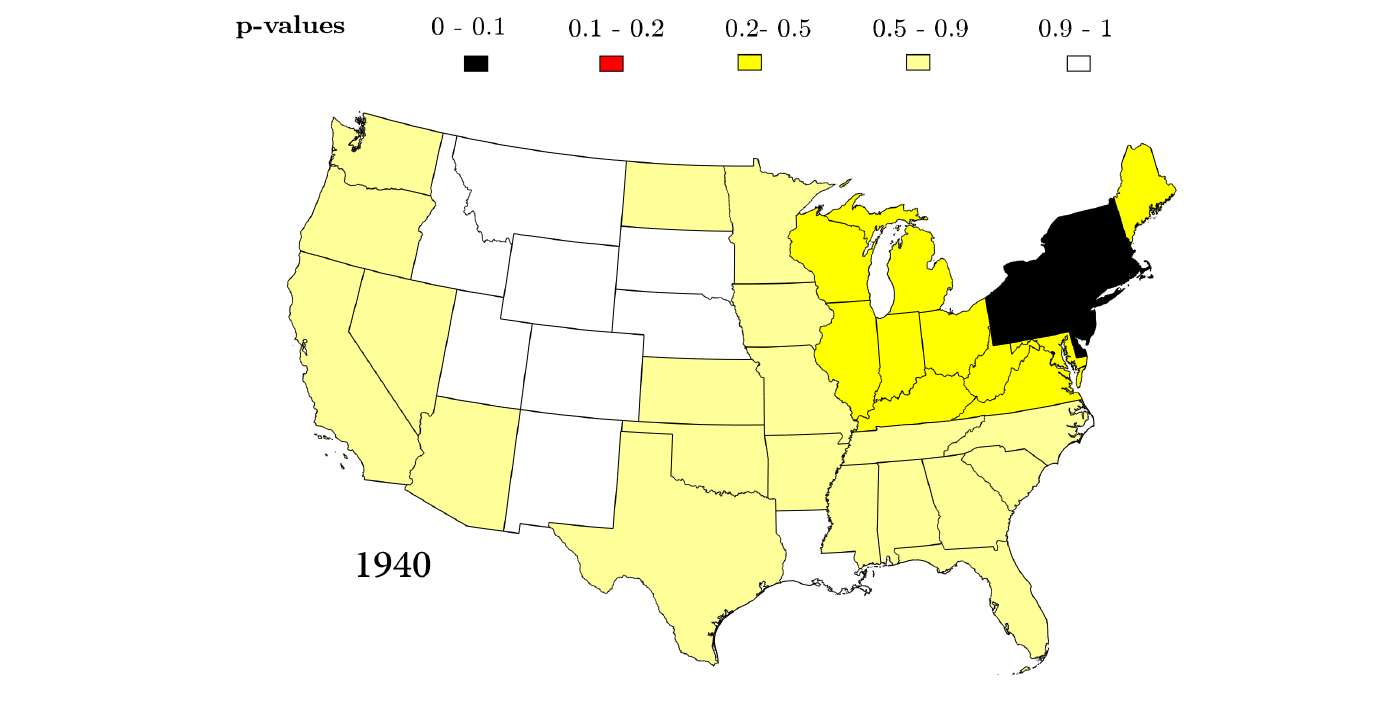

Supplement: S7 Fig — The colored areas reflect the significance (p-value) of local concentration of social influence for each state. The p-values for each state are derived from a random permutation test of local clustering using the Getis-Ord Local Gi* statistic (see Fig 5 in main text for details). (TIF) [file pone.0177970.s009.tif]

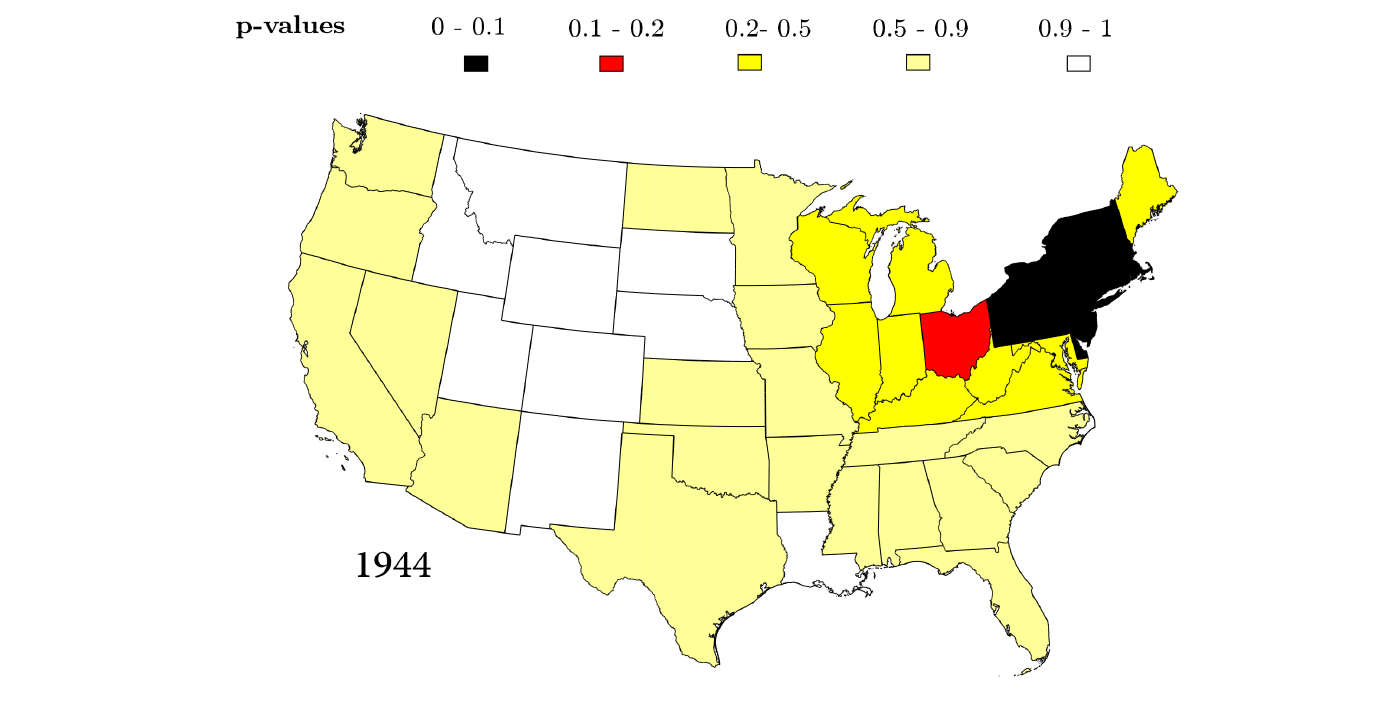

Supplement: S8 Fig — The colored areas reflect the significance (p-value) of local concentration of social influence for each state. The p-values for each state are derived from a random permutation test of local clustering using the Getis-Ord Local Gi* statistic (see Fig 5 in main text for details). (TIF) [file pone.0177970.s010.tif]

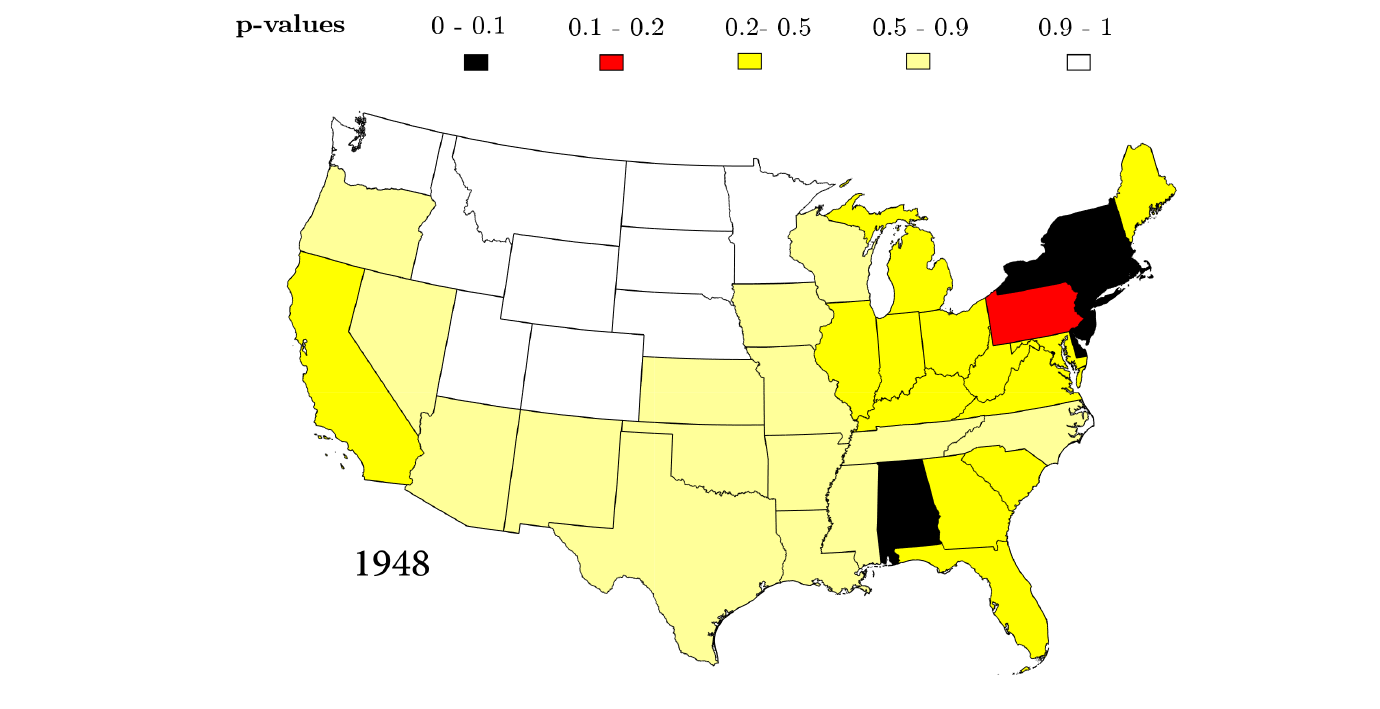

Supplement: S9 Fig — The colored areas reflect the significance (p-value) of local concentration of social influence for each state. The p-values for each state are derived from a random permutation test of local clustering using the Getis-Ord Local Gi* statistic (see Fig 5 in main text for details). (TIF) [file pone.0177970.s011.tif]

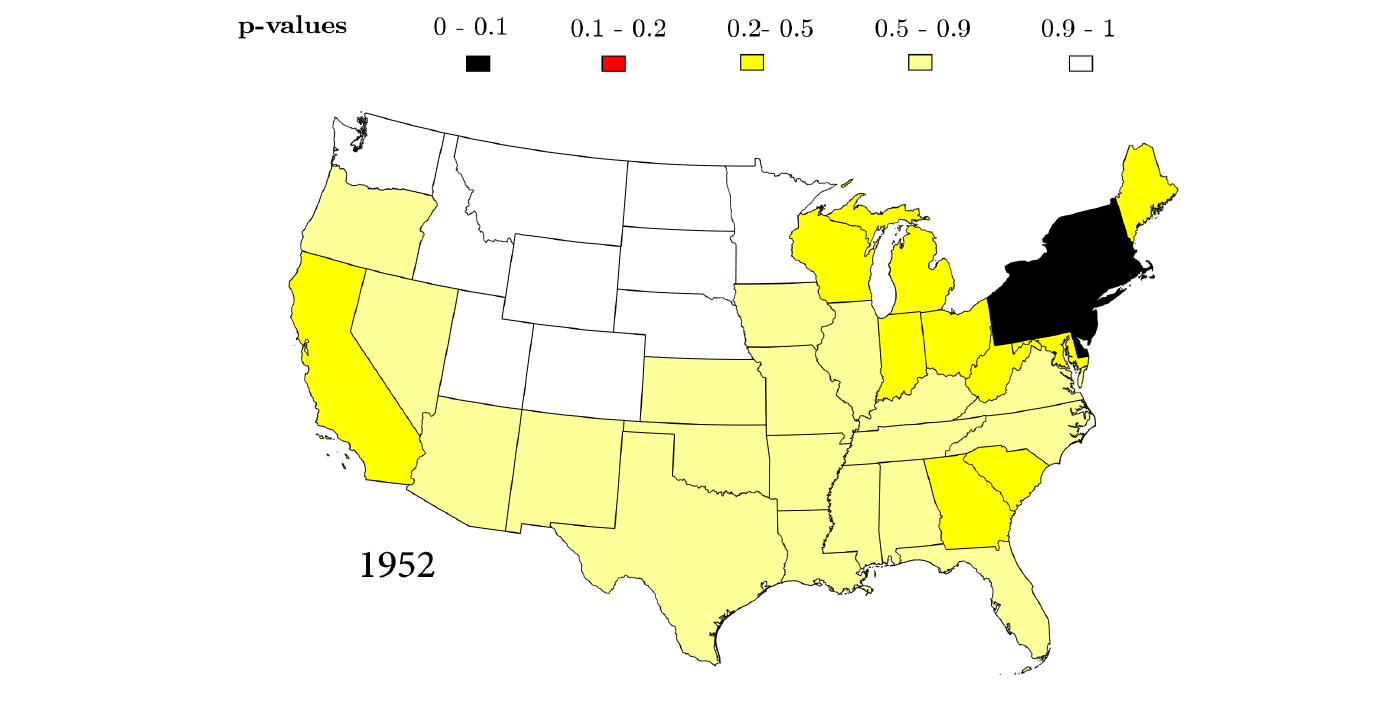

Supplement: S10 Fig — The colored areas reflect the significance (p-value) of local concentration of social influence for each state. The p-values for each state are derived from a random permutation test of local clustering using the Getis-Ord Local Gi* statistic (see Fig 5 in main text for details). (TIF) [file pone.0177970.s012.tif]

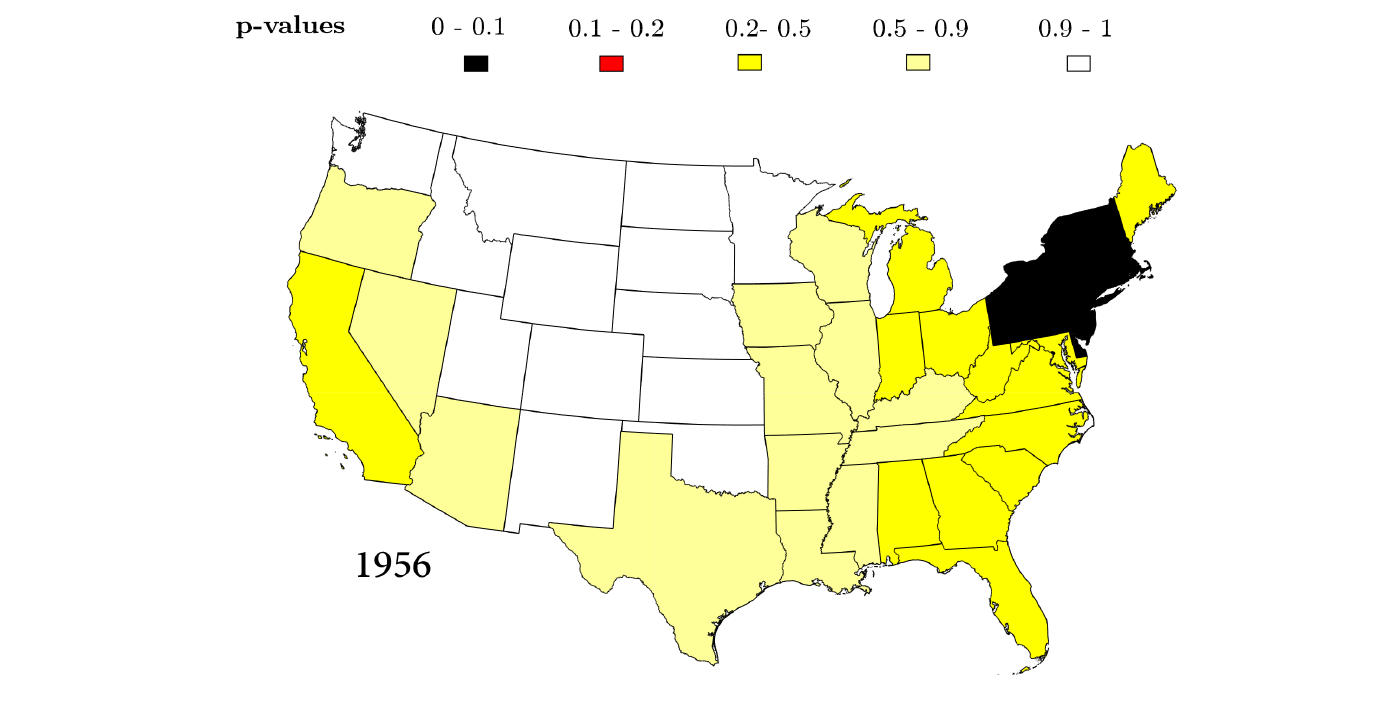

Supplement: S11 Fig — The colored areas reflect the significance (p-value) of local concentration of social influence for each state. The p-values for each state are derived from a random permutation test of local clustering using the Getis-Ord Local Gi* statistic (see Fig 5 in main text for details). (TIF) [file pone.0177970.s013.tif]

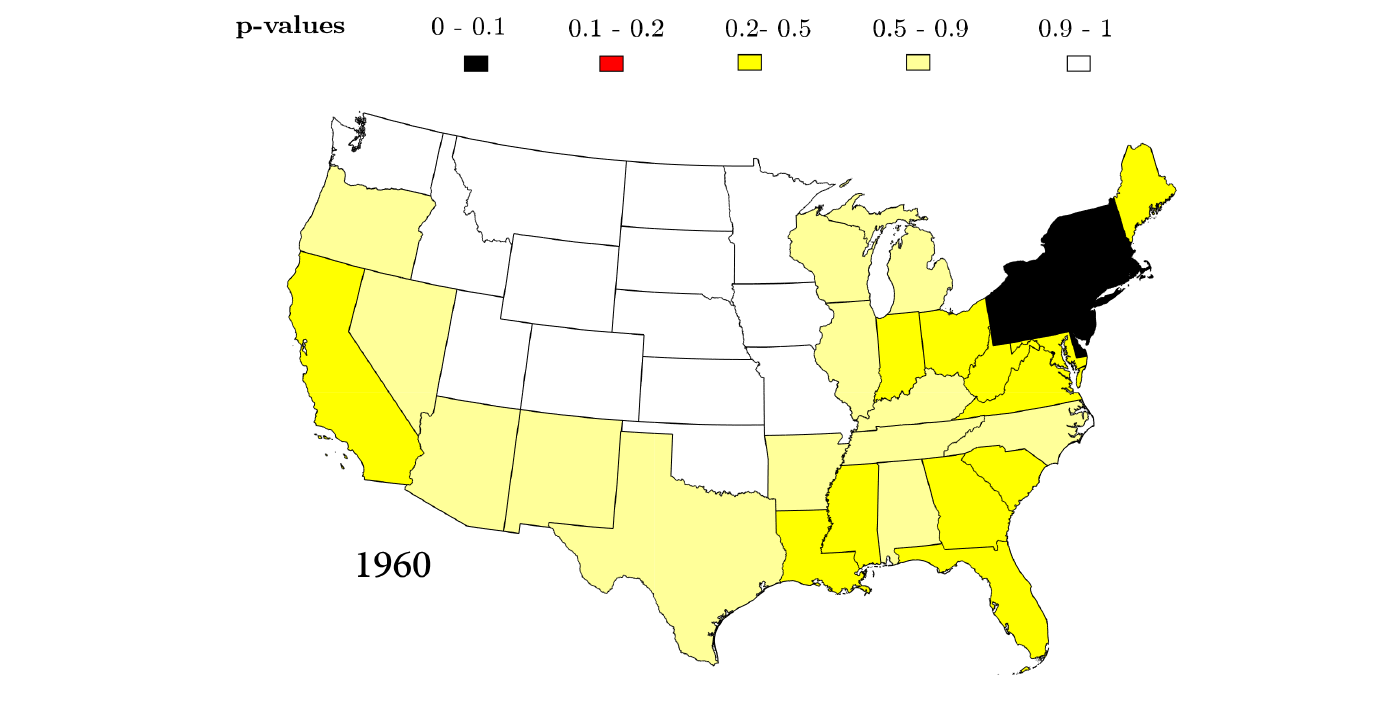

Supplement: S12 Fig — The colored areas reflect the significance (p-value) of local concentration of social influence for each state. The p-values for each state are derived from a random permutation test of local clustering using the Getis-Ord Local Gi* statistic (see Fig 5 in main text for details). (TIF) [file pone.0177970.s014.tif]

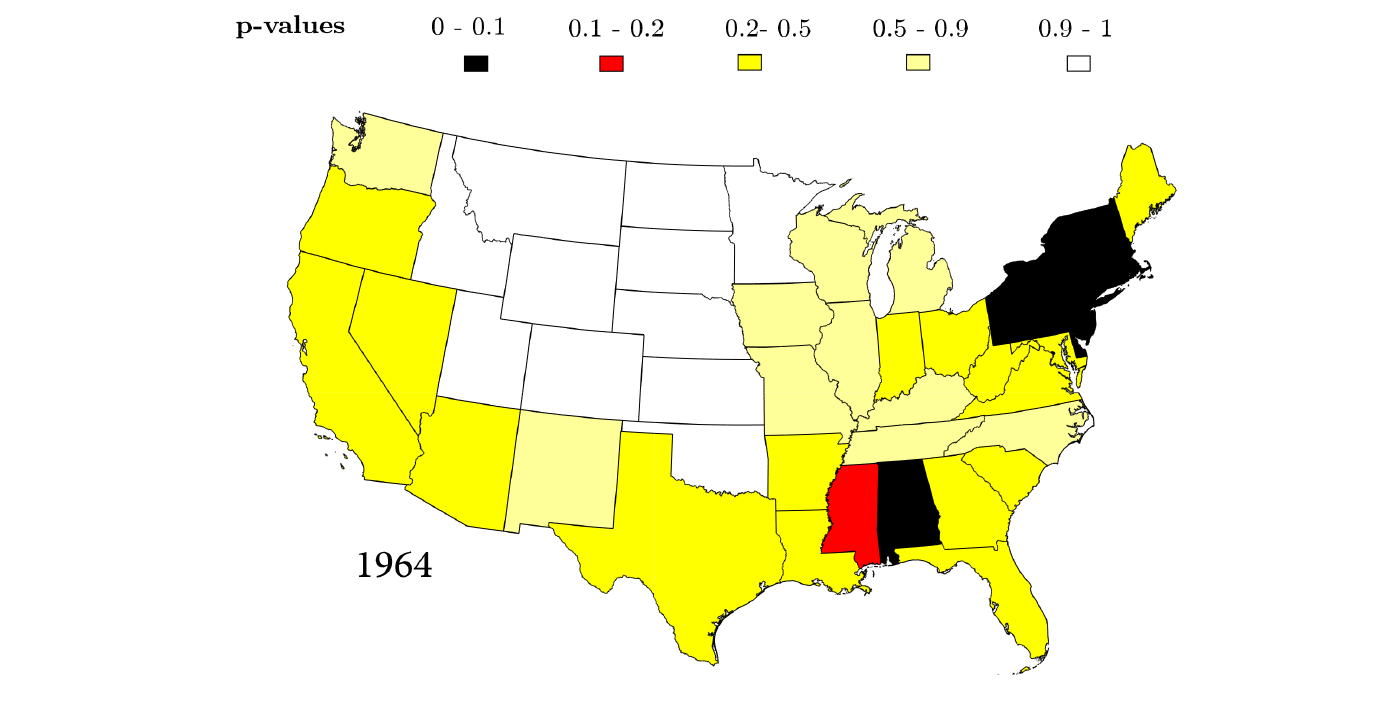

Supplement: S13 Fig — The colored areas reflect the significance (p-value) of local concentration of social influence for each state. The p-values for each state are derived from a random permutation test of local clustering using the Getis-Ord Local Gi* statistic (see Fig 5 in main text for details). (TIF) [file pone.0177970.s015.tif]

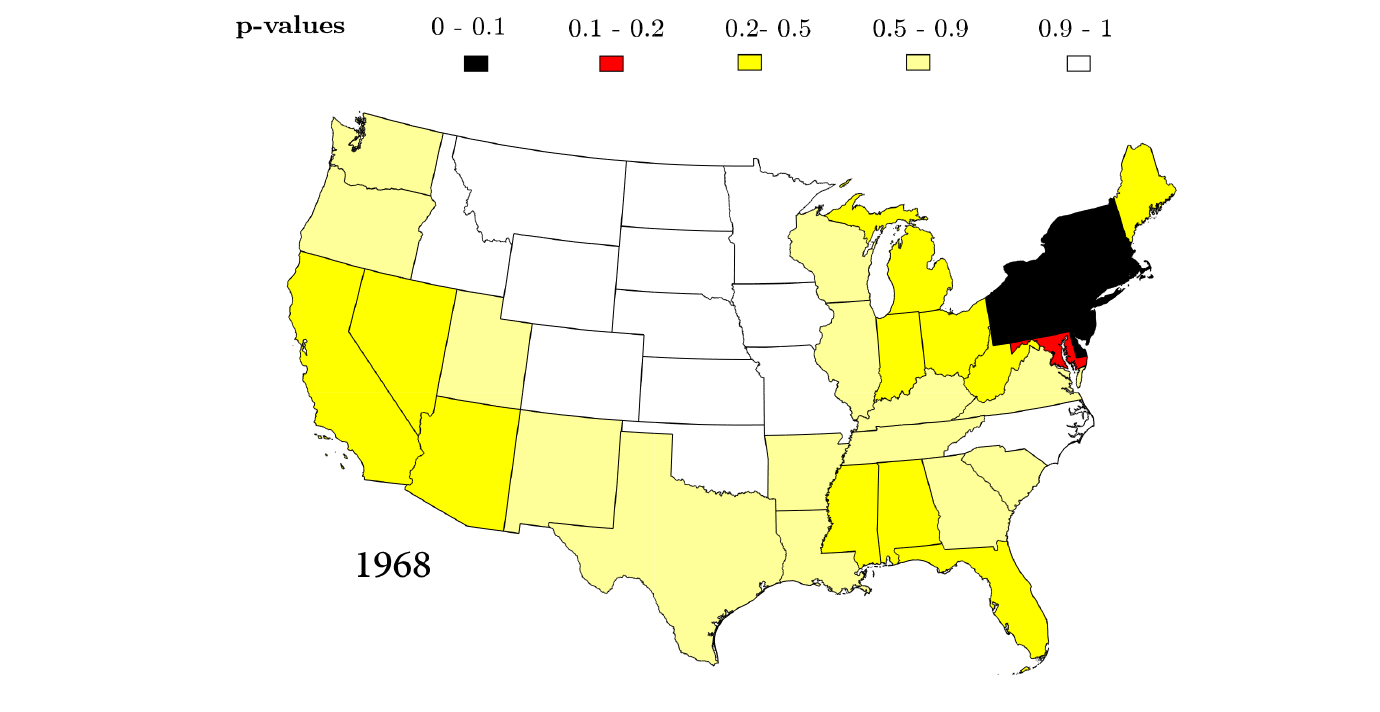

Supplement: S14 Fig — The colored areas reflect the significance (p-value) of local concentration of social influence for each state. The p-values for each state are derived from a random permutation test of local clustering using the Getis-Ord Local Gi* statistic (see Fig 5 in main text for details). (TIF) [file pone.0177970.s016.tif]

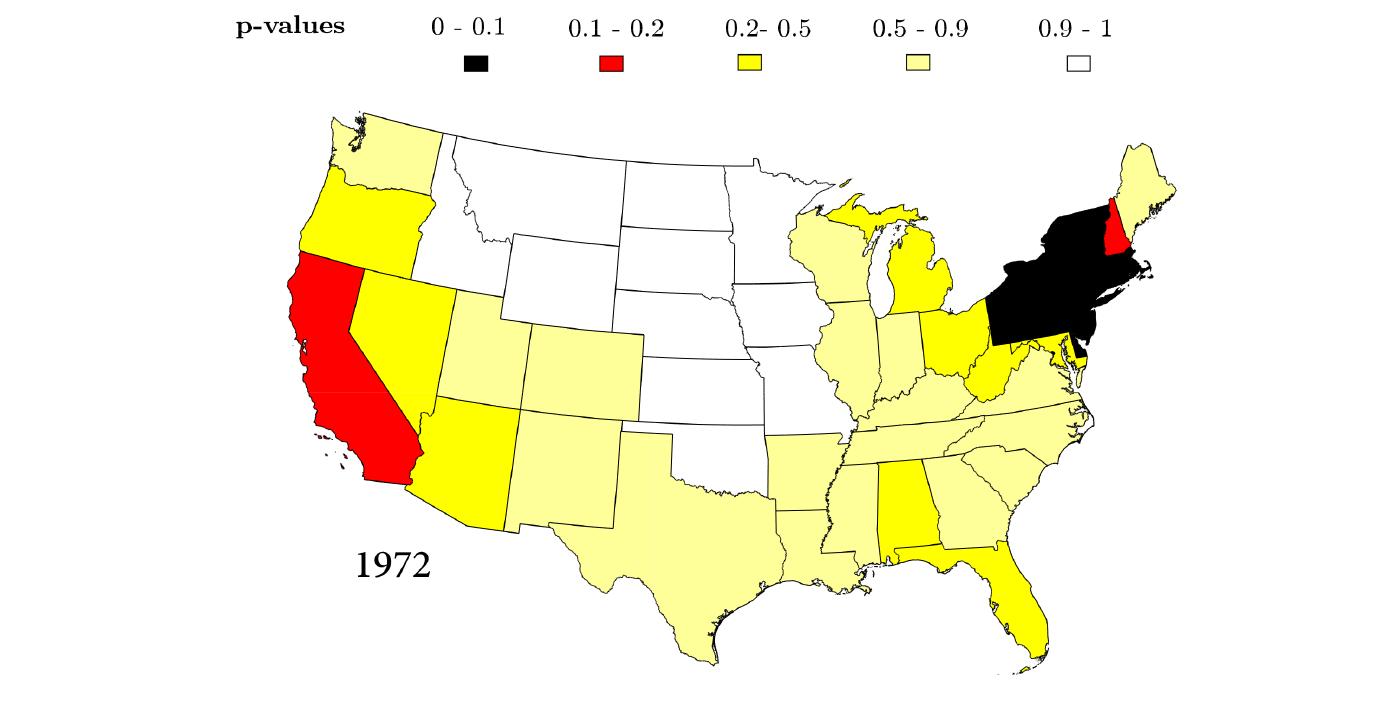

Supplement: S15 Fig — The colored areas reflect the significance (p-value) of local concentration of social influence for each state. The p-values for each state are derived from a random permutation test of local clustering using the Getis-Ord Local Gi* statistic (see Fig 5 in main text for details). (TIF) [file pone.0177970.s017.tif]

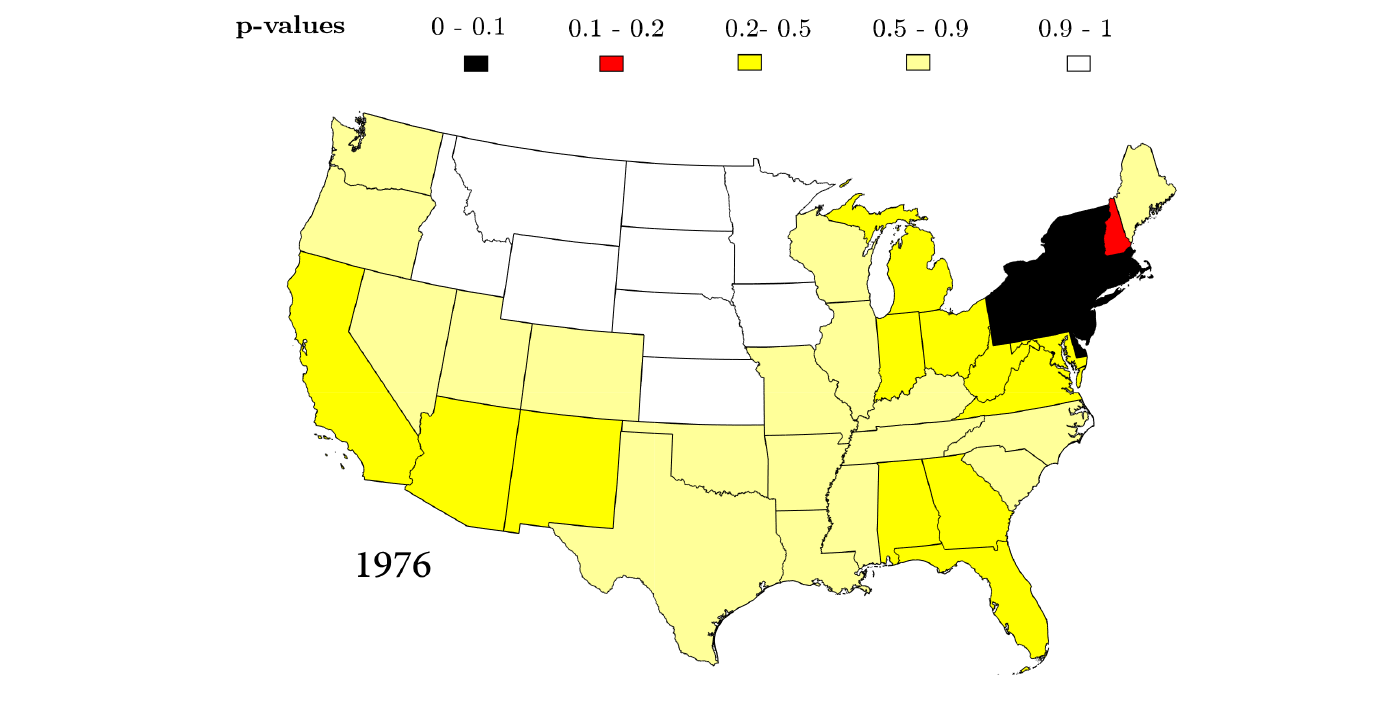

Supplement: S16 Fig — The colored areas reflect the significance (p-value) of local concentration of social influence for each state. The p-values for each state are derived from a random permutation test of local clustering using the Getis-Ord Local Gi* statistic (see Fig 5 in main text for details). (TIF) [file pone.0177970.s018.tif]

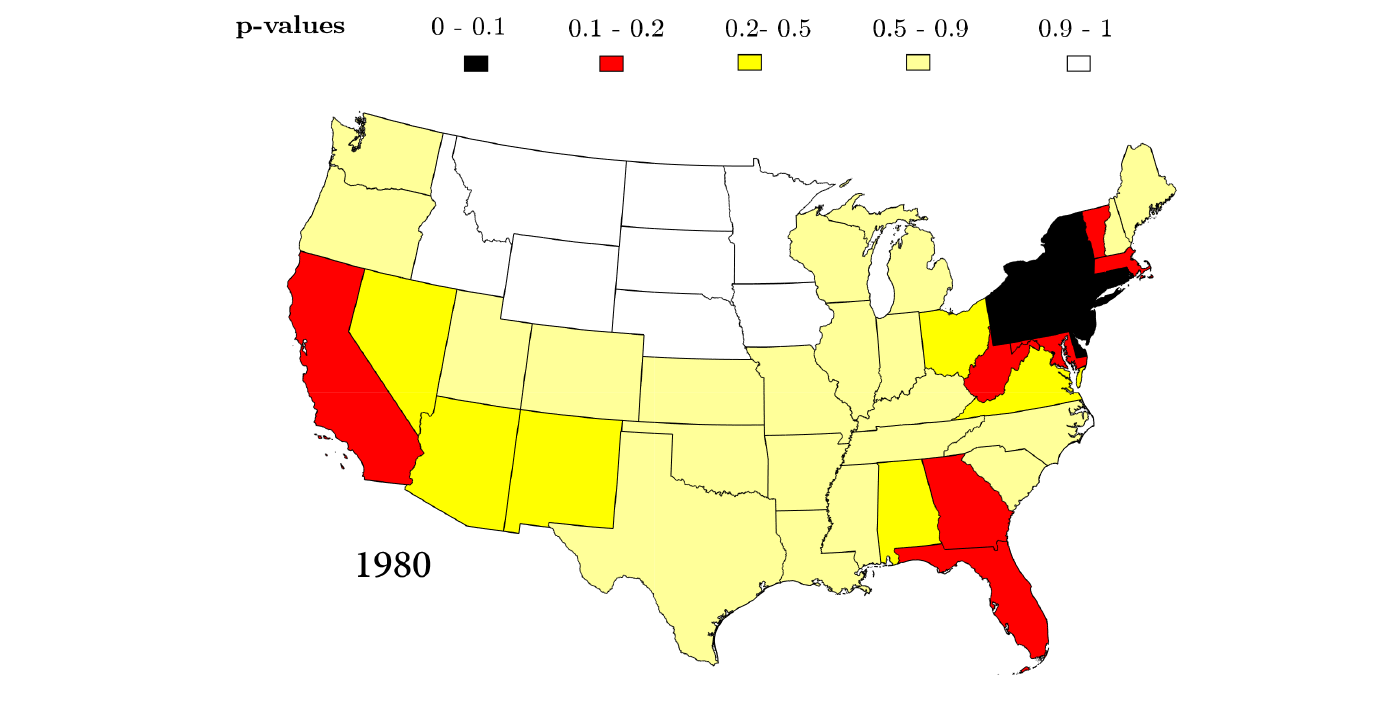

Supplement: S17 Fig — The colored areas reflect the significance (p-value) of local concentration of social influence for each state. The p-values for each state are derived from a random permutation test of local clustering using the Getis-Ord Local Gi* statistic (see Fig 5 in main text for details). (TIF) [file pone.0177970.s019.tif]

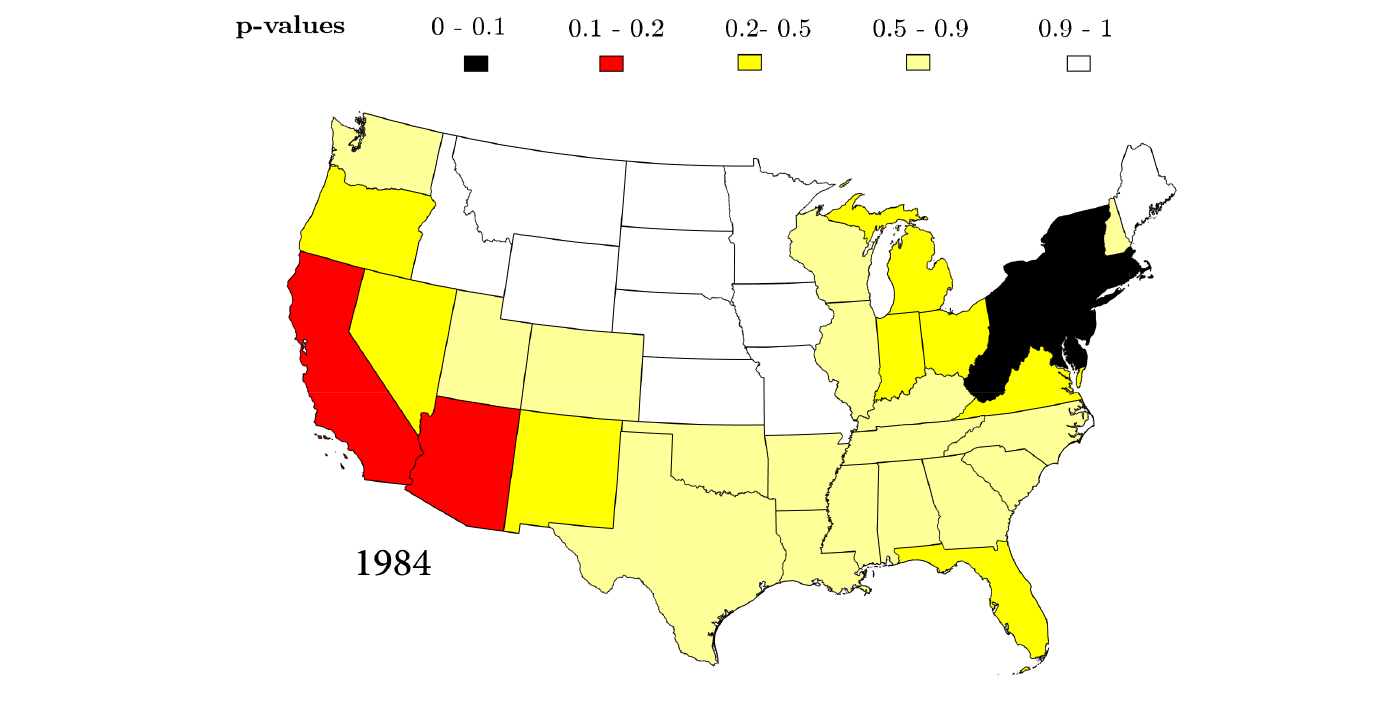

Supplement: S18 Fig — The colored areas reflect the significance (p-value) of local concentration of social influence for each state. The p-values for each state are derived from a random permutation test of local clustering using the Getis-Ord Local Gi* statistic (see Fig 5 in main text for details). (TIF) [file pone.0177970.s020.tif]

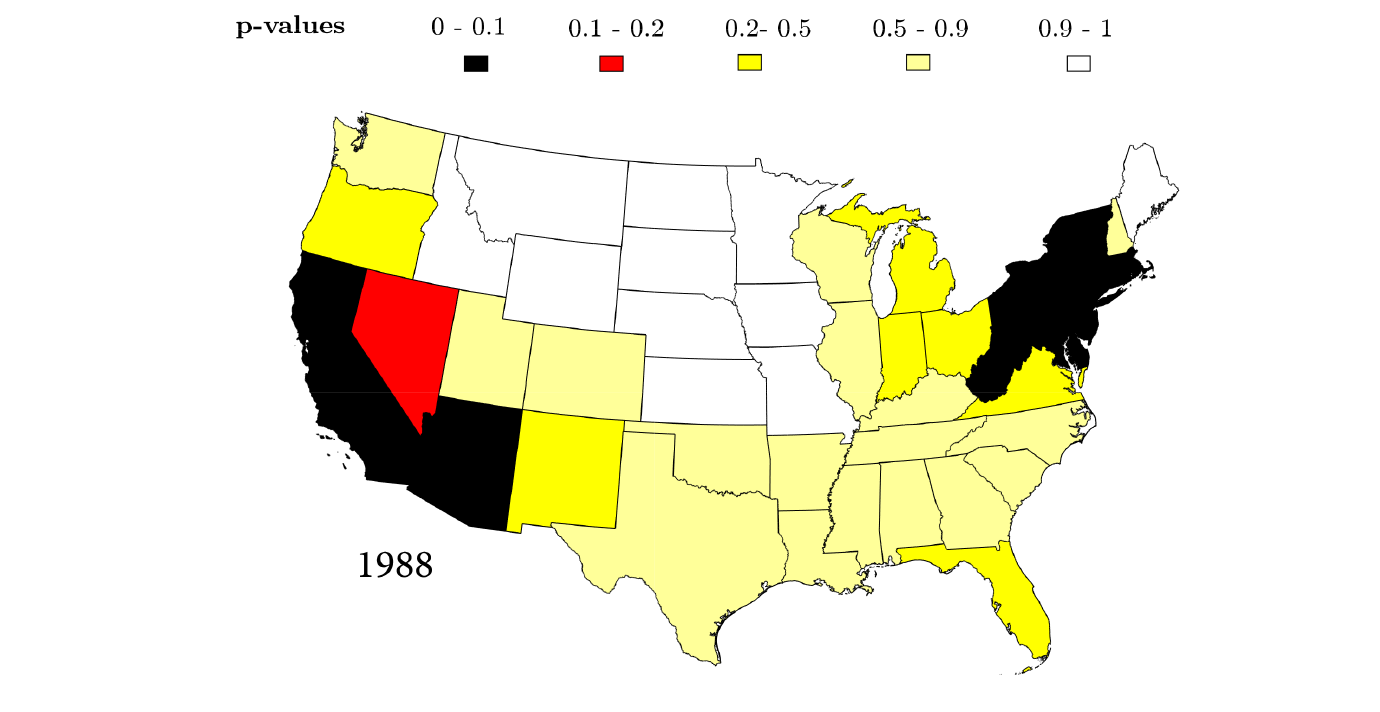

Supplement: S19 Fig — The colored areas reflect the significance (p-value) of local concentration of social influence for each state. The p-values for each state are derived from a random permutation test of local clustering using the Getis-Ord Local Gi* statistic (see Fig 5 in main text for details). (TIF) [file pone.0177970.s021.tif]

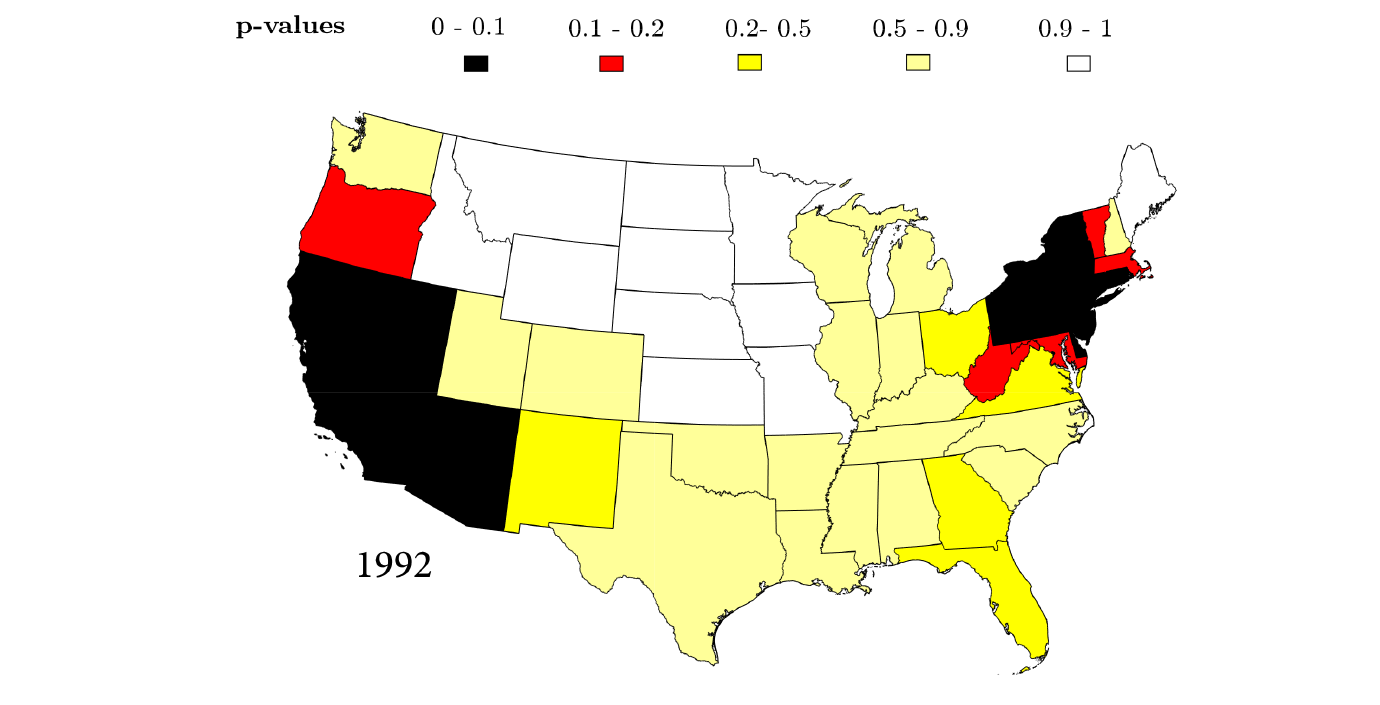

Supplement: S20 Fig — The colored areas reflect the significance (p-value) of local concentration of social influence for each state. The p-values for each state are derived from a random permutation test of local clustering using the Getis-Ord Local Gi* statistic (see Fig 5 in main text for details). (TIF) [file pone.0177970.s022.tif]

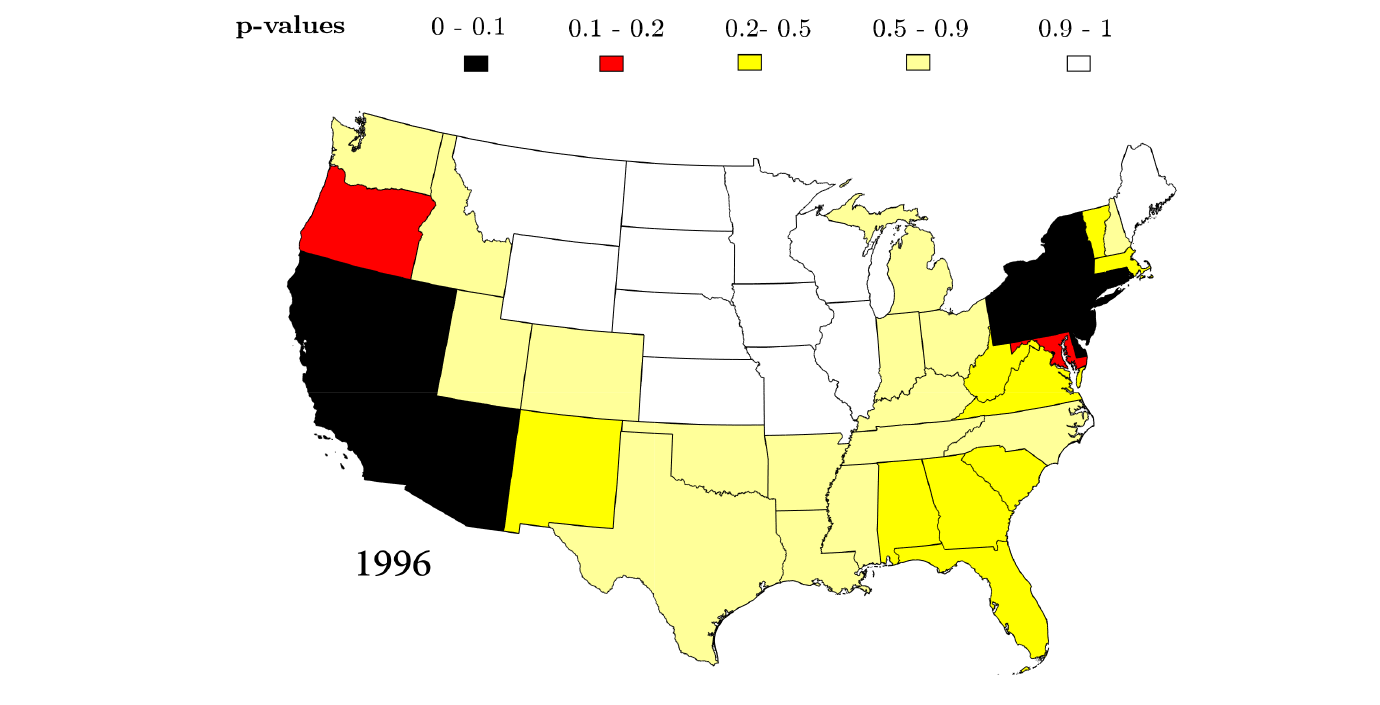

Supplement: S21 Fig — The colored areas reflect the significance (p-value) of local concentration of social influence for each state. The p-values for each state are derived from a random permutation test of local clustering using the Getis-Ord Local Gi* statistic (see Fig 5 in main text for details). (TIF) [file pone.0177970.s023.tif]

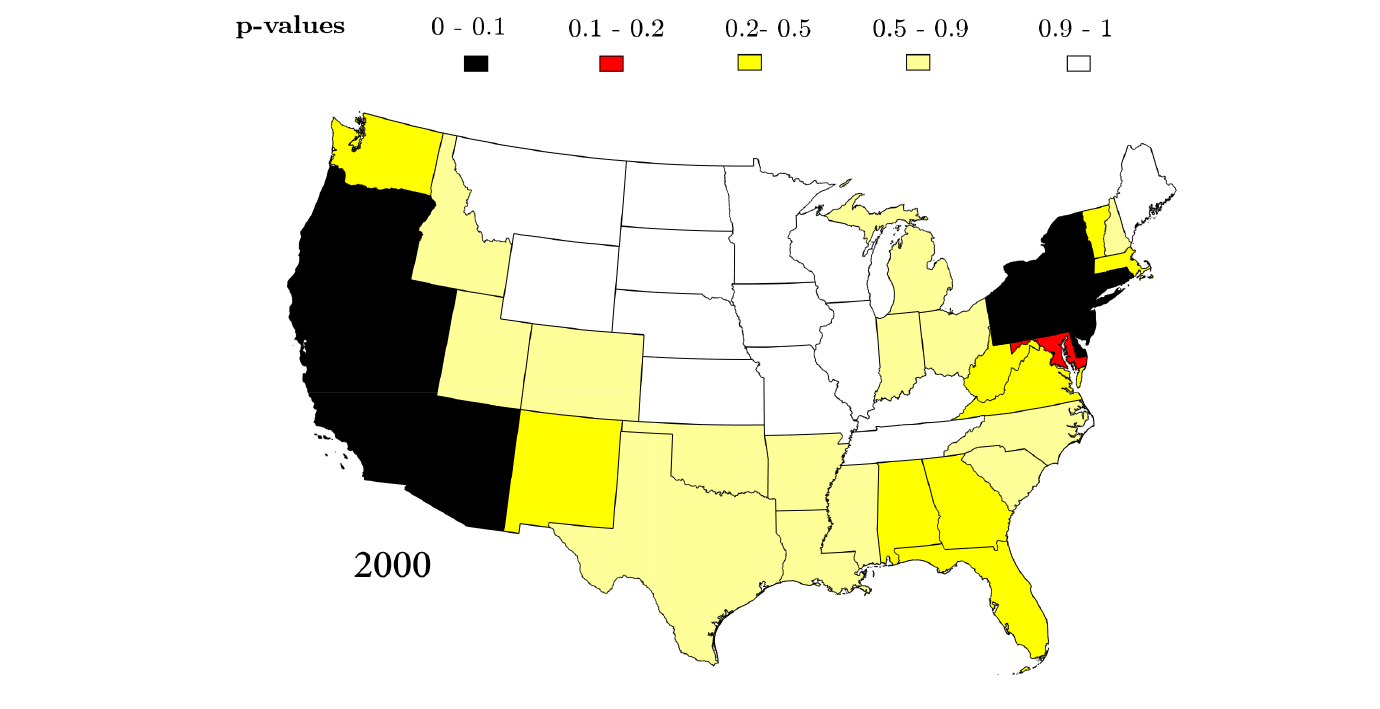

Supplement: S22 Fig — The colored areas reflect the significance (p-value) of local concentration of social influence for each state. The p-values for each state are derived from a random permutation test of local clustering using the Getis-Ord Local Gi* statistic (see Fig 5 in main text for details). (TIF) [file pone.0177970.s024.tif]

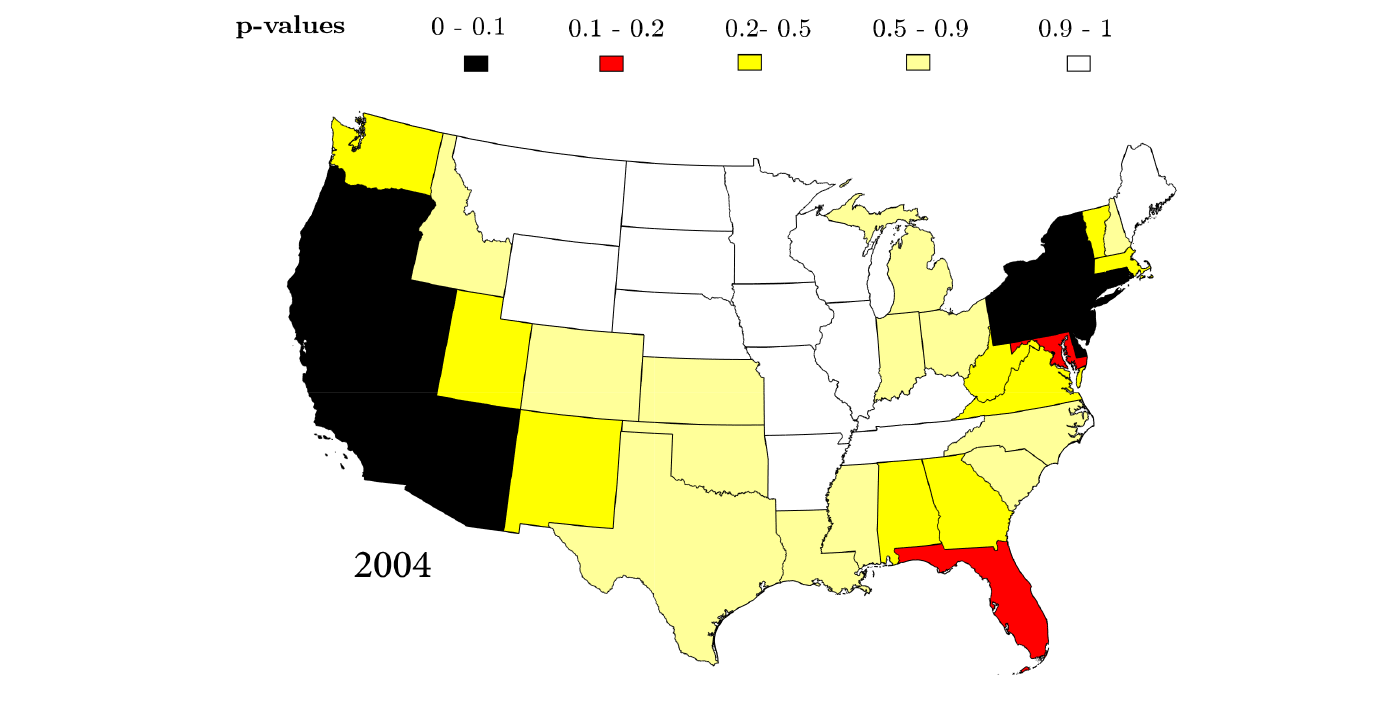

Supplement: S23 Fig — The colored areas reflect the significance (p-value) of local concentration of social influence for each state. The p-values for each state are derived from a random permutation test of local clustering using the Getis-Ord Local Gi* statistic (see Fig 5 in main text for details). (TIF) [file pone.0177970.s025.tif]

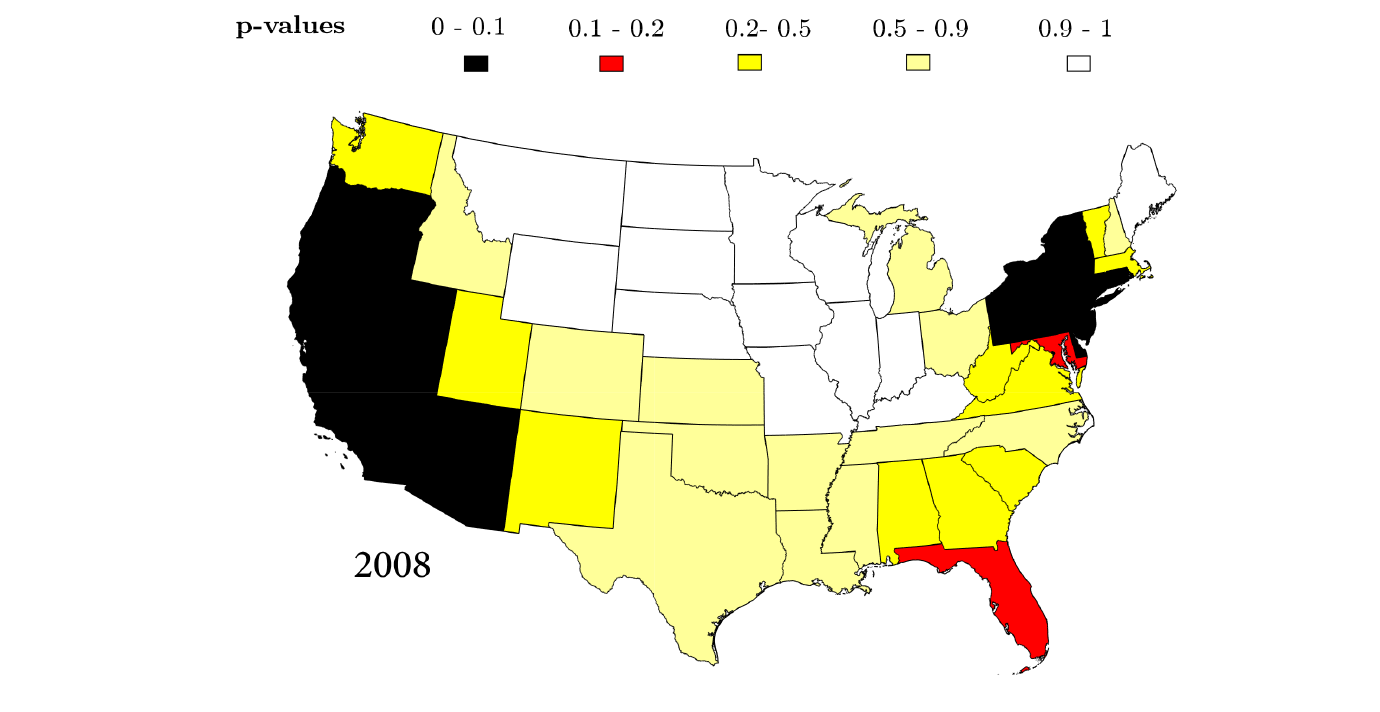

Supplement: S24 Fig — The colored areas reflect the significance (p-value) of local concentration of social influence for each state. The p-values for each state are derived from a random permutation test of local clustering using the Getis-Ord Local Gi* statistic (see Fig 5 in main text for details). (TIF) [file pone.0177970.s026.tif]

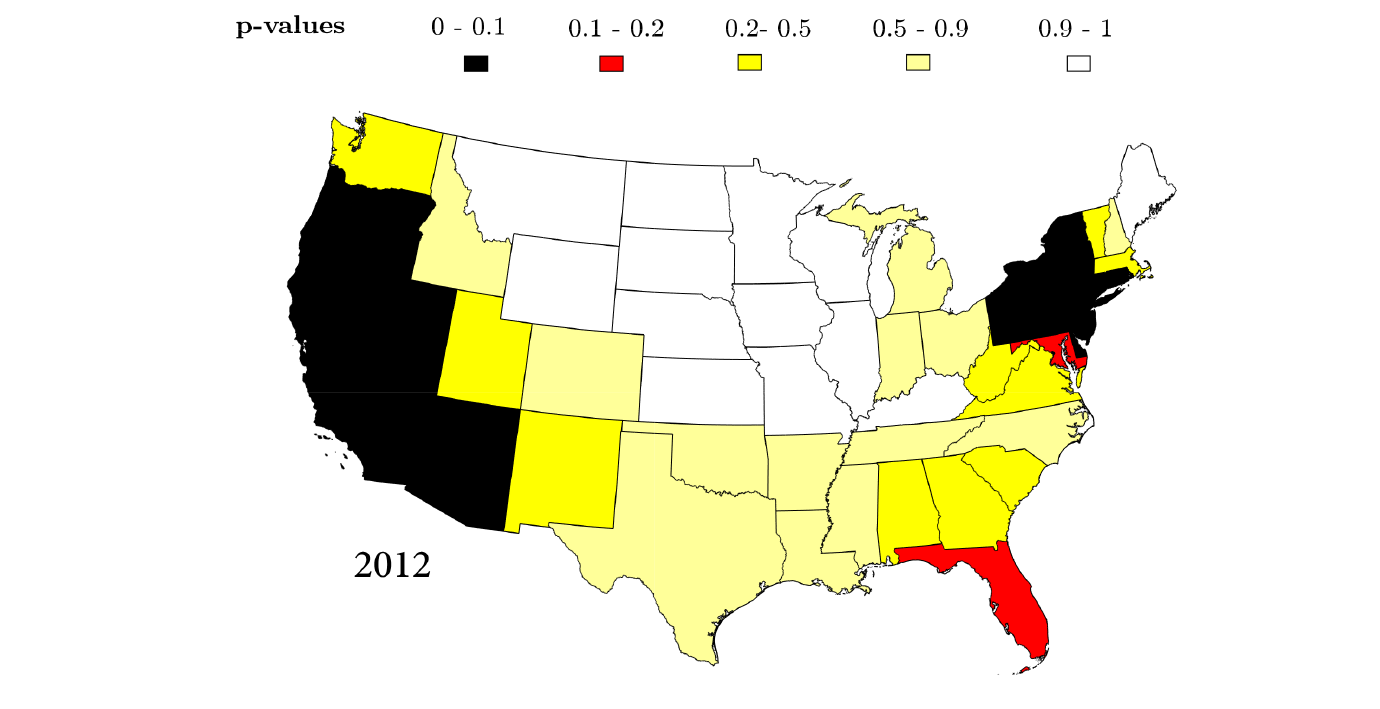

Supplement: S25 Fig — The colored areas reflect the significance (p-value) of local concentration of social influence for each state. The p-values for each state are derived from a random permutation test of local clustering using the Getis-Ord Local Gi* statistic (see Fig 5 in main text for details). (TIF) [file pone.0177970.s027.tif]

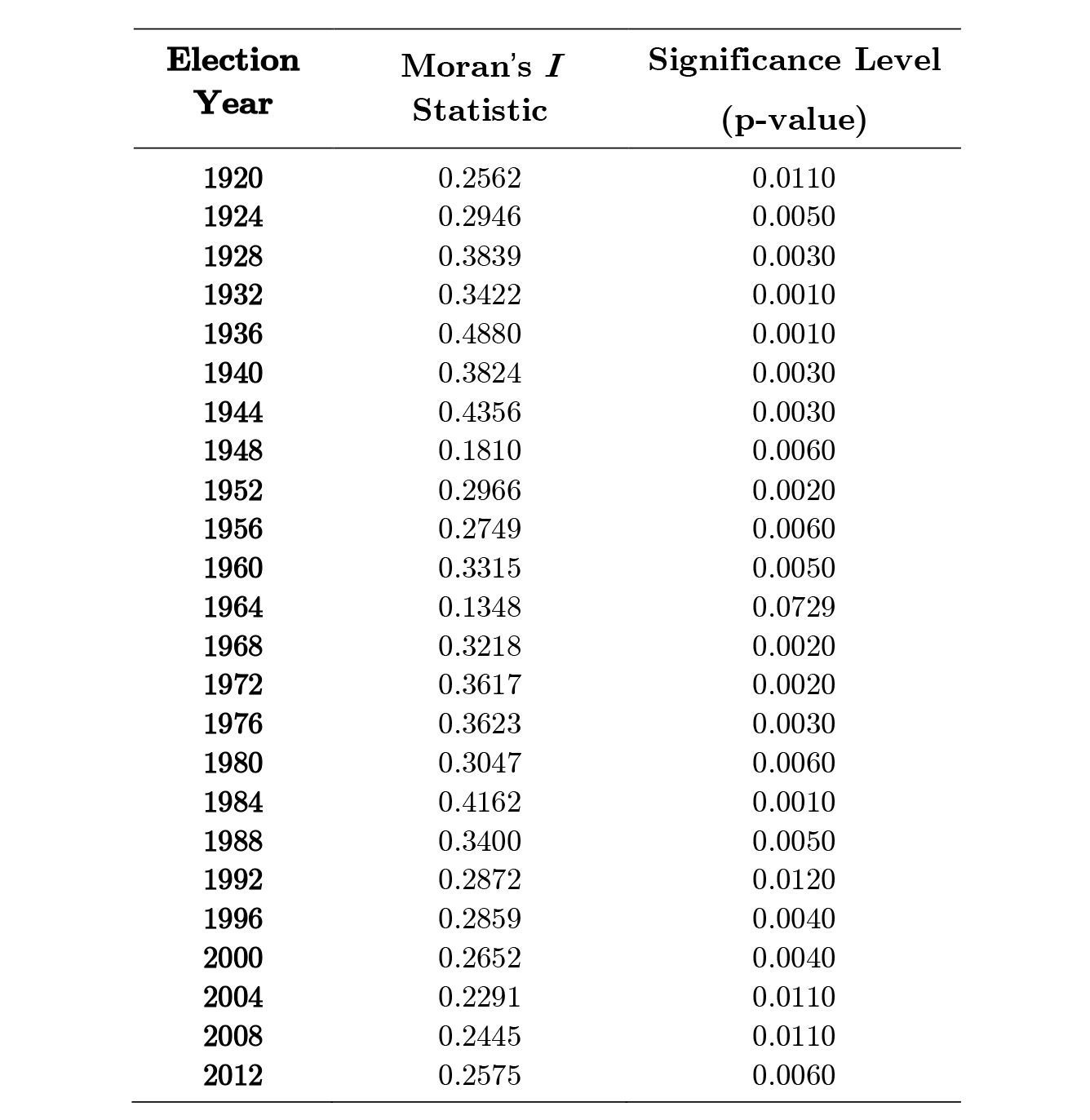

Supplement: S1 Table — This analysis was performed with a contiguity spatial weight matrix (row normalized) that indicates whether states share a boundary or not. The variable of concern is the social influence index calculated using Eq 7 in the main text. The observed Moran’s I statistics are shown in the second column and the corresponding significance levels (p-values) of the tests are shown in the third column. The random permutation tests suggest the presence of significant positive spatial autocorrelation as indicated by the level of significance (p-value) shown in the third column. (TIF) [file pone.0177970.s028.tif]

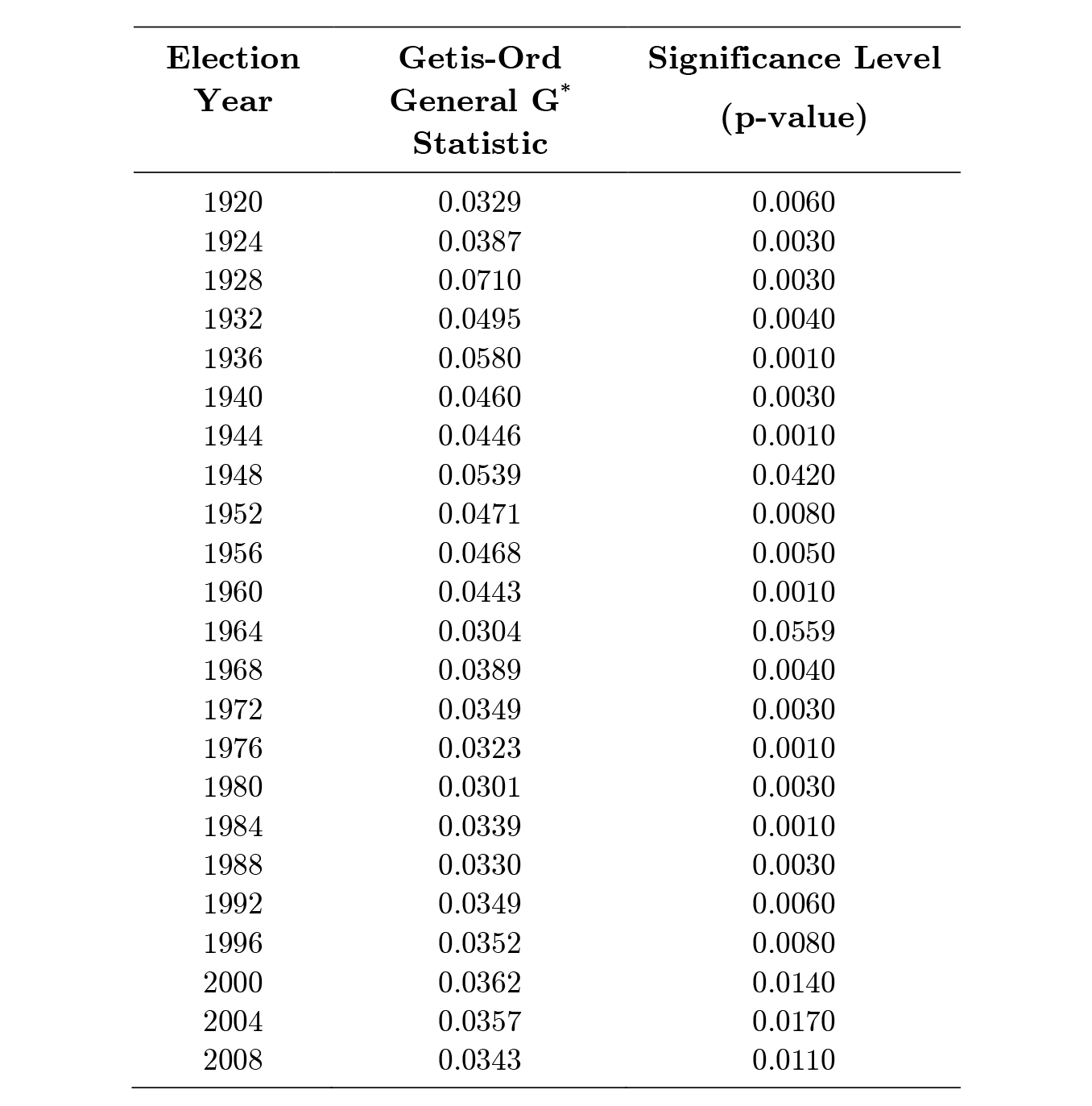

Supplement: S2 Table — This analysis was performed with a contiguity spatial weight matrix that indicates whether states share a boundary or not. The variable of concern is the social influence index calculated using Eq 7 in the main text. The observed Getis-Ord General G* statistics and significance levels (p-values) of the tests are shown in the second and third columns, respectively. The tests indicate that social influence is significantly concentrated in space as shown by the significance levels (p-value) in the third column. For all election years, the observed Getis-Ord General G* is larger than the expected General G*, indicating that the spatial distribution of high social influence values is more spatially clustered than would be expected if underlying spatial processes were truly random. (TIF) [file pone.0177970.s029.tif]
